# Dielectrophoresis Tutorial: Inspired by Hatfield's 1924 Patent and Boltzmann's Theory and Experiments of 1874

**DOI:** 10.1002/elps.8114

**Published:** 2025-03-06

**Authors:** Ronald Pethig

**Affiliations:** ^1^ School of Engineering, Institute for Integrated Micro and Nanosystems University of Edinburgh Edinburgh UK

**Keywords:** dielectrophoresis, electrostatics, electrokinetics, particle separation, ponderomotive force

## Abstract

The first patent to describe dielectrophoresis (DEP) as a means and process to separate particles from a mixture was granted by the US Patent Office to Henry Stafford Hatfield in 1924. The novel methods of sample preparation and designs of electrode geometry covered by the patent's disclosures and claims describe the basis for most present‐day applications of DEP as a method of particle separation. Hatfield had clearly acquired a deep understanding of DEP, not only from the perspective of the potential energy of the target particle to be separated from others but also from consideration of the conservation of energy of the electrical system. He cites no prior art or supporting theory of what he would have known as the action of the ponderomotive force. There is no record of his publishing this work, but efforts to find the source of his knowledge led to the retrieval of his Ph.D. thesis from the archives of the University of London. It describes innovative applications of DEP to separate impurity particles from powdered mineral ore and refers to the theoretical and experimental studies by Boltzmann of the ponderomotive force acting on small spheroidal samples of insulating material. This early background theory and experimentation by Boltzmann is described here and, together with the rules and methods so clearly and simply described in Hatfield's patent, forms the core of a tutorial for those engaged in DEP but who might lack formal training in physics.

## 1 Introduction

The motivation and encouragement for submitting this article evolved during the DEP2024 Conference in Dublin. This event took place 100 years almost to the day after the patent “Means and Process of Separating Substances One from Another” [1] was granted to Henry Stafford Hatfield, and 150 years after Ludwig Boltzmann's investigations of the ponderomotive force acting on dielectric particles in an electric field gradient [2, 3]. The patent clearly describes applications of dielectrophoresis (DEP)—the term created by Pohl [4]—but which Hatfield would have known as the action of the ponderomotive force investigated by Boltzmann [2, 3] as cited by Drude [5] and referenced in his Ph.D. thesis [6]. Drude mostly describes ponderomotive forces associated with the magnetic field. Generations of German students acquired their understanding of the electric ponderomotive force from Föpple's textbook “*Introduction to Maxwell's Theory of Electricity*” [7] and later editions of it coauthored with Abraham [8, 9]. The basic “rules” for particle manipulation and separation by DEP that Hatfield so clearly describes [1, 6] and understood 100 years ago remain valid and instructive today. DEP is an interdisciplinary research field and so it was felt that a significant number of the conference attendees could benefit from the descriptive rules given by Hatfield (and largely overlooked by the DEP community). A member of the organizing committee of the conference, who serves as editor‐in‐chief of electrophoresis, kindly invited me to contribute an article for the Conference's Special Issue to highlight this 100th anniversary for DEP and as a tutorial. This implied the straightforward aim of extracting Hatfield's “rules” from his patent and as a related objective to uncover the source of his deep understanding of electrostatic field theory not to be found, for example, from texts by Faraday, Maxwell, or Thomson.

### 1.1 Hatfield: A UK Citizen in Germany

Hatfield was influenced by texts published in German. This is evident not just from the style of his “rules” of DEP but also from a snippet of his remarkable life story. Boltzmann (1844–1906), whose tombstone at the Zentralfriedhof in Vienna bears his entropy equation S = k logW, requires no such snippet. In a patent application [10] submitted when he was 20 years old, Hatfield cites his occupation as an electrochemist and student at University College London (UCL). As an undergraduate, he was the personal research assistant to William Ramsay (1904 Nobel Prize in Chemistry) [11]. He graduated from UCL in 1902 with a B.Sc. in chemistry and 1922 with a Ph.D. [12]. In 1903 he was appointed by an English company to manage the production of their electricity meters, for which special glass from Germany was required [13]. This was the borosilicate glass developed by the Schott company in Jena, and as explained in his thesis this led him there [6]. In seven patents granted to him in the years leading up to the First World War (July 1914), Hatfield describes himself as a citizen of Great Britain, with addresses in Jena and then Braunschweig. From 1914 to 1918, he was interned at Ruhleben—a converted horseracing venue near Berlin [14]—where he founded its Arts & Science Union and organized lectures in chemistry and engineering up to London University degree standard [14, pp. 148–152]. By 1916 a reference library of around 5000 books had been created at Ruhleben for this intellectual activity [14, p. 153] and this most likely included the Abraham‐Föppl textbooks.

### 1.2 The Abraham‐Föpple Textbooks

According to Holton [15], Föppl's textbook was widely bought—particularly because of the author's ability to present Maxwell's theory clearly to engineers. Einstein “buried himself” into it “with a certain fanaticism day and night” [15, p. 153]. In his foreword, Föppl explains that German physicists, unlike their English counterparts, were slow to be convinced by Maxwell's field theory of electricity because they were still caught up in the spell of action at a distance [7]. Their initial efforts were directed toward the reconciliation of the two theories. But then scientifically educated technicians and teachers of science in Germany sought to acquaint themselves with the basic features of Maxwell's theory. Föppl invited Max Abraham (who had studied under Max Planck) to co‐author the 2nd edition and by 1907 the foreword to the 3rd edition [8] could state that a new one was necessary because “the number of physicists, technicians, and mathematicians who are interested in Maxwell's theory is growing from year to year”. As evidence of its popularity with teachers and students, seven editions of “Abraham & Föppl” were printed by the time of Max Abraham's death in 1922 (August Föppl died in 1924). The chapter titled “The Energy and Ponderomotive Forces of the Electrostatic Field” [8, pp. 167–185] would certainly have been useful to Hatfield as a teaching aid.

### 1.3 Aim and Limits of this Tutorial

**FIGURE 1:**
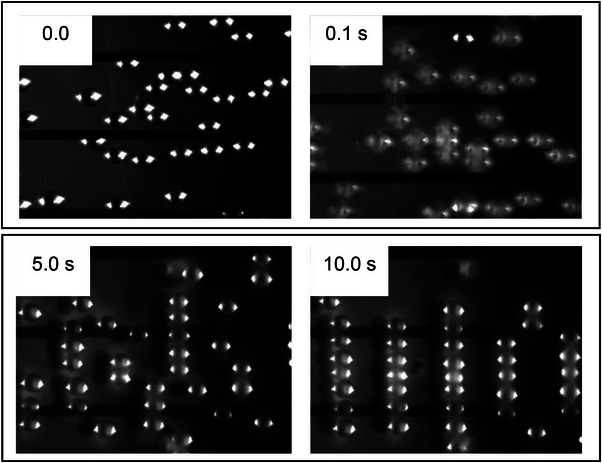
The timed DEP response of latex beads suspended in an aqueous medium above an interdigitated array of electrodes (unpublished images related to Markx et al. [16]).

Consider the time sequence shown in Figure 1 of the DEP response of latex beads in an aqueous medium, initially at rest on an array of coplanar interdigitated electrodes. At time zero in Figure 1 an AC signal (2‐V pk‐pk, 1 MHz) was applied to the electrodes. In response, within 1 s the beads have levitated above the plane of the electrodes—a manifestation of the DEP force at work. This was followed by the formation of chains of beads. Hatfield would have recognized this DEP response, and particle chaining, as an induced polarization of nonconducting particles and the action of a ponderomotive force to increase the dielectric capacity of the system. In his mind, he probably formed an intuitive model to explain this effect from the viewpoint of the particle. Within a dielectric particle, or at the surface of a metal particle, the separation of positive and negative electrical charges (+*q* and −*q*) over a small distance is induced by the applied electric field **E**. If the field is inhomogeneous the forces +*q* **E**_1_ and −*q* **E**_2_ acting at each end of this induced dipole are of different strengths. Unless constrained to do so, the particle moves to minimize its potential energy. But Hatfield also viewed the effect from the point of view of the system.

Work is done by the voltage signal generator to create charge density distributions on the electrodes. Energy is then stored in the generated field **E**, which is uniquely defined when the charge density distribution and dielectric constant of the medium are known throughout the system. The time 0 and 10 s views of the system shown in Figure 1 are quite different. Viewing what happens from the point of view of the particle is achieved using a microscope. However, viewing what happens from the point of view of energy conservation of the system is another matter and not often considered by the DEP community. But through the conservation of energy approach lies the promise of automatic monitoring and control of the DEP manipulation of particles.

Hatfield was not trained as a physicist but, through the theorems taught by the Drude and Abraham‐Föppl textbooks, he clearly became a master of DEP theory and practice. The aim of this tutorial is to provide, for those interested in DEP but who lack training in physics or electromagnetics, an insight into the theory of electrostatics that inspired Hatfield 100 years ago and was known to Boltzmann 50 years before that. Those mainly attracted by a “hands‐on” approach can skip the theory and go straight to section 5 which describes Hatfield's patent and the contents of his Ph.D. thesis recently retrieved from the archives of London University.

## 2 Boltzmann: Theory and Experiments

Boltzmann [2] was motivated by the remarkable consequence, apparently not recognized by Clausius, Helmholtz, and Maxwell, of the behavior of electric insulators in an electric field. The electric field must exert a considerable force on a nonconductor without itself being electrified, but instead by virtue of its induced dielectric polarization. A small sphere of insulating material having a dielectric constant *ε*, that is electrified only by induction, will experience a force in free space some [(ε−1)/(ε+2)] times that of a small metal sphere of equal size and similarly electrified only by induction. Boltzmann realized that measurement of the ponderomotive (i.e., DEP) force acting on a polarized particle in a nonuniform field can be used to determine its dielectric constant. As defined by Faraday an induced polarization manifests itself as a “particle being positive on one side and negative on the other, the positive and negative electrification of each side being always exactly equal” [17, p. 57]. The net charge of the particle remains zero. The degree of polarization reflects the magnitude of this induced dipole moment per unit volume of the particle.

Boltzmann's experiments employed the torsion balance, first devised by Michell and used by Cavendish to determine the weak gravitational force between two solid bodies. It was reinvented by Coulomb in 1784 to discover his eponymous law of force between bodies that carry an electric charge [18, pp. 204–220]. A torsion balance consists of a horizontal arm suspended by either a filament of metal wire or an insulating fiber such as silk. The body to be examined is attached to one end of the arm, counterbalanced by a mirror at the other end. The arm is free to swing about the vertical axis of the suspending filament. A force acting in a tangential direction to the test body causes the filament to twist by an angle that can be determined using a graduated scale. The moment of inertia of the balancing arm is first calculated and a calibration is made of the torsional rigidity of the filament. The force of attraction or repulsion acting on the test body is then determined by focusing a telescope on the mirror to determine the frequency of free oscillation of the arm about the twist angle.

### 2.1 Theory

Drude [5] summarizes the theory, formulated by Boltzmann and published in *Reports of Sessions of the Imperial Academy of the Sciences and Humanities of Vienna* [e.g., 3] as follows: Consider a nonconducting spherical body, composed of an isotropic material of dielectric constant *ε*, located in an insulating medium of dielectric constant *ε*_m_ in which an electric field E has been established. The force, **F**_x_, acting on a volume element dτ of this body along the *x* coordinate is given by Equation (1) [5, p. 293]:

(1) $$F_{x}=\frac{\varepsilon -\varepsilon_{m}}{8\pi }\cdot \frac{\varepsilon_{m}}{\varepsilon }\cdot \frac{\partial E^{2}}{\partial x}d\tau .$$

Similar formulae apply for the *y*‐ and *z*‐axes. Now, if the factors $\partial E^{2}/\partial x,\;\partial E^{2}/\partial y$ and $\partial E^{2}/\partial z$ were to remain constant throughout the whole sphere of the dielectric, then these three formulae would directly indicate the components of the forces acting on the sphere. However, this assumption is not permitted no matter how small the volume of the sphere may be. However, if the sphere is small enough so that the flow of the electric lines of force within the sphere is almost the same as if it were in a uniform field, this task can be solved exactly [5, p. 49]. It follows from this that the force acting on the whole sphere when located in free space (i.e., *ε*_m_ = 1) has the value (Equation 2):

(2) $$F=\left[\frac{\left(\varepsilon -1\right)}{4\pi \left(1+\frac{\varepsilon -1}{3}\right)}\right]\frac{\partial E^{2}}{\partial x}V=\frac{3}{4\pi }\left[\frac{\left(\varepsilon -1\right)}{\left(\varepsilon +2\right)}\right]\frac{\partial E^{2}}{\partial x}V,$$

where *V* is the volume of the sphere. This is the now familiar DEP force equation expressed in the cgs system of units (to convert to SI units replace 1/4π with *ε*_o_ the electric constant). Analogous equations are obtained for the force components $\partial E^{2}/\partial y,\;\partial E^{2}/\partial z.$ The three components of force can now be determined by exchanging the insulating sphere with a conductive sphere of the same size. The easiest way to do this is to cover the insulating sphere with a very thin gold leaf. Hollow metallic or material‐filled spheres act in an electrostatic field the same way as a solid metallic sphere. A conductive sphere also acts like a sphere of an insulator, whose dielectric constant is infinitely large [5, p. 268]. The effect on the metallic ball in the electric field therefore follows by assigning to *ε* the value of infinity in Equation (2):

(3) $$F^{\prime }=\frac{3}{4\pi }\frac{\partial E^{2}}{\partial x}V.$$

If the spheres are small, they will influence the flow of the electric lines of force only in their immediate vicinity, and the factors $\partial E^{2}/\partial x$ etc will be almost the same outside the spheres in both cases. If, therefore, $\partial E^{2}/\partial x$ in Equations (2) and (3) are equated with each other, the coefficient [(ε−1)/(ε+2)] is obtained by comparing the values of **F** and **F**'.

Boltzmann extended his theory to consider the consequence of the nonuniform field being created by an electrified macroscopic sphere and not a point charge and calculated the forces acting on nonspherical samples [3].

### 2.2 Experiments

**FIGURE 2:**
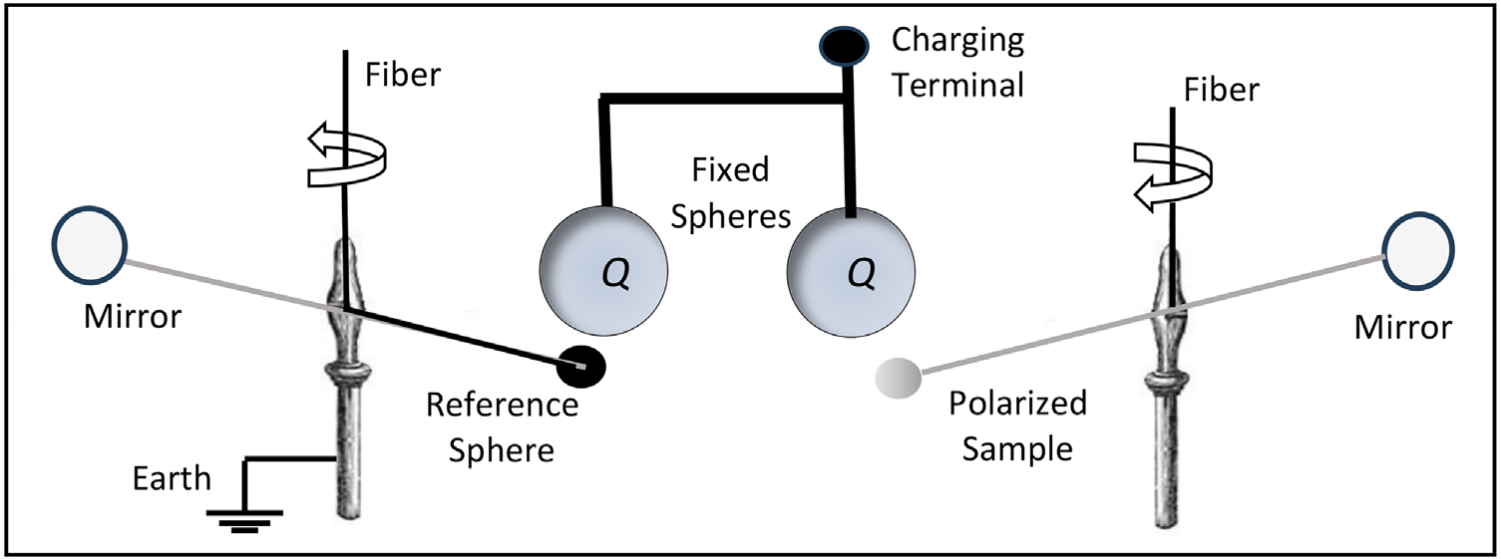
Boltzmann used a torsion balance to measure the ponderomotive force acting on a polarized sample and a second one to monitor the magnitude of the charge *Q*.

Boltzmann [2] carried out his experiments in the laboratory of August Töplar in Graz, with the assistance of Albert von Ettingshausen, using the Töplar “influence” machine [19, p.96] to electrify a fixed metallic sphere located near a much smaller test sphere attached to one end of the horizontal arm of a torsion balance. The electrified sphere generated the nonuniform field required to produce the ponderomotive force given by Equation (2). Töplar's machine‐generated electrostatic energy by friction and consisted of two glass disks, one fixed and the other rotating. The fixed glass disk was said to influence the rotating one by inducing charges on its surface. The large metal sphere was electrified by allowing a spark to bridge a gap between the discharge terminal of the Töplar machine and the charging terminal connected to the sphere. Although the spark gap remained the same, it was not possible to always supply the same electric charge *Q* to the fixed ball. To correct for this inconsistency the fixed metal sphere was electrically connected to another one, as shown in Figure 2, and a second balance was used to measure the force exerted on a reference metal sphere. This reference sphere was electrically connected to Earth. The reading taken for the right‐hand balance was divided by that for the left‐hand balance and used as a multiplying factor for each measurement of the attractive force acting on the polarized spherical sample being investigated for its value of dielectric constant. This sample was then covered with gold leaf so that it behaved as a solid metal sphere. Since it was always necessary to look through two telescopes at the same time, Boltzmann required Ettingshausen's assistance—“with no small sacrifice of time and effort” [2, p. 534]. (In 1886 with his Ph.D. student Walther Nernst (1920 Nobel Prize in Chemistry) they discovered the Ettingshausen‐Nernst Effect—a thermoelectric phenomenon where a transverse thermal gradient is developed in a conductor carrying electric current flow if subjected to a magnetic field.)

The whole arrangement shown in Figure 2 was enclosed inside a Faraday cage of gold foil. Careful checks were made that neither the fiber to the horizontal arm from which the test sphere was hung, nor the components in the cage, were affected and that the rotation of the torsion arm could only arise from the test insulating sphere being influenced by the charge *Q* on the fixed sphere. The fact that the test sphere was neither previously nor perceptibly electrified continuously was established by alternately charging the fixed sphere several times positively and negatively. The insulating sphere was then replaced by a conductive sphere of the same size to compare the effect that both would experience. The value of the dielectric constant of the test material was derived by comparing the quotient of these two readings with the theoretical ratio of [(ε−1)/(ε+2)]. This procedure was repeated five times for each of the four dielectric materials investigated to give the average values shown in Table 1. Also included in Table 1 are the dielectric constant values obtained by Boltzmann with other experiments [2, 3] based on Faraday's capacitor method and also by measurement of its refractive index. The latter method was based on Maxwell's electromagnetic theory of light, which gives the dielectric constant of a transparent medium equal to the square of its index of refraction [17 (vol 2, p. 437), 20].

**TABLE 1:** Values of the dielectric constant obtained by Boltzmann's measurements of the ponderomotive force, Faraday's capacitance method, and Maxwell's equation for the optical refractive index.

	Boltzmann	Faraday	Maxwell
Sulfur	3.94	3.84	4.06
Paraffin	2.32	2.32	2.33
Pine resin	2.48	2.55	2.38
Hard rubber	3.48	3.15	—

As shown in Table 1, close agreement of the dielectric constants was obtained by Boltzmann from the three methods of measurement.

Boltzmann refers [2] to further experiments, where a wire leading to the electrified sphere was attached to a vibrating tuning fork so that it made contact more than 100 times a second to the positive and just as often to the negative electrode of the Töplar influence machine. In this way, the small sphere to be examined was rapidly alternated from positive to negative induced electrification. The attractive force of the dielectric spheres which resulted from this was found to be consistent with that determined by DC electrification and in agreement with the theory for the cases of hard rubber, paraffin, and pine resin (colophonium). Boltzmann also investigated the effect on the ponderomotive force of changing the spherical shape of the test sample to that of an ellipsoid caused by a change in the distribution of induced surface charges [3]. He also investigated how the dielectric constant varied with the orientation of the principal axes of a sulfur crystal [21].

## 3 Electrostatics Theory for DEP

Föppl [7, pp. 1–4] judged there to be nine essential aspects of Maxwell's doctrine to be taught. The first two of these are:

- All electric and magnetic action of one body on another distant one is transmitted through the space between them, whether that space is empty or filled with matter.
- The seat of electric or magnetic energy is to be found not only in the electrified or magnetized body but also to a much greater extent in the surrounding field.

The ninth essential characteristic that Föppl considered a student should learn was:

- The representation of mathematical relations by equations involving vectors.

Föppl explains that although Maxwell occasionally presented his equations according to Hamilton's quaternion theory, he mainly employed the Cartesian mode of representation. In Föppl's opinion, this obscured the faithful description of Faraday's theories of the flow of electric force. “Vector calculus must be used, and the effort it takes to familiarize oneself with it is amply outweighed by the advantages gained”. Part 1 of his book is thus devoted to *Vectors and Vector Fields*, followed by: Part 2 (*The Electric Field*); Part 3 (*The Magnetic Field*); Part 4 (*Further Expansion of the Theory*). This format is retained in all eight editions of Föppl & Abraham, with Parts 1 and 2 remaining virtually constant in content, with significant changes made by Abraham in Part 4 reflecting the continuing advances of the subject. An important chapter in Part 2, and of relevance to present developments of DEP, is the final one titled *The Energy and Ponderomotive Forces of the Electrostatic Field* [8, pp. 167–185], where the ponderomotive force F*_p_* acting on a particle is defined in terms of a spatial gradient of its potential energy *U* (Equation 4):

(4) $$F_{P}=-\frac{\partial \left(U-U_{0}\right)}{\partial x}=-\nabla U_{P}.$$

The minus sign in this equation indicates that the particle is driven toward a location in the field where the particle's free energy (electrochemical potential) is reduced. This definition is sometimes employed now but is more likely to be presented as the force acting on a particle's induced dipole moment **m** in a field gradient ∇E:

(5) $$F_{DEP}=m\cdot \frac{\partial E}{\partial x}=\left(m\cdot \nabla \right)E$$

Equations (4) and (5) are equivalent because the potential energy of a rigid dipole aligned along the direction of **E** is given by Equation (6):

(6) $$U=-\left(m\cdot E_{x}\right).$$

The induced moment **m** is rigid in the sense that it is always aligned along the direction of the local applied field. For the case of a particle (e.g., a protein) that possesses a permanent dipole moment by virtue of its intrinsic molecular structure, its “tumbling” and resulting torque in a field needs also to be considered. Work done (energy gained) on displacing a particle having a rigid induced moment is the product of the force applied over a distance. Equations (4) and (5) are thus connected through a relationship that sums up (integrates) the incremental changes of potential energy along the total path taken by the particle, expressed as Equations (7):

(7) $$U_{DEP}=-\int F_{DEP}.ds.$$

Equation (5) equates the DEP force to (m·∇)E, which is a vector field function involving a vector ‘dot’ product and the nabla “operator” ∇. For those for whom it would be helpful, a brief revision follows of the vector algebra used as stepping stones of the path leading to Equations (2) and (4).

### 3.1 Vectors

Föppl adopts the vector algebra developed by Hamilton (1853) as well as Maxwell's use of Gothic (old German) symbols to denote a vector (“the number of different vectors being so great that Hamilton's favorite symbols would have been exhausted at once” [17, vol II, p.257]). Vectors of arbitrary direction and magnitude can be specified by their components in fixed directions. For this purpose, three mutually perpendicular unit vectors **i**, **j**, **k** are chosen whose directions are those of the axes of a Cartesian co‐ordinate system. Hamilton's algebra involves the quaternions (*a, b, c, d*) corresponding to *a* + *b*i + *c*j + *d*k, with i, j, k identified as three distinct square roots of −1 such that ij = k, jk = i, ki = j and jk = −kj, kj = −i, ik = −j. The symbol “*a*” represents the “real” one‐dimensional “scalar” part of the quaternion. If the quaternions are portrayed geometrically, any unit vector in the i, j, k space is also a square root of −1, so that this space has the homogeneity required for a representation of ordinary three‐dimensional space [17, vol 2, pp. 257–261]. The quaternions no longer play a central role in physics, partly because they have been reformulated as the Pauli matrices which, together with the unit matrix, have the same algebraic structure as the quaternions. For example, they are the representation of Lie algebra (conceived by Sophus Lie in the 1870s) and occur in the Pauli equation, which considers the interaction of electron spin with an electromagnetic field [22]. The term “scalar” adopted by Hamilton does, however, remain in use. The last of the various vector algebra formulas presented by Abraham & Föppl [5, p. 115] is expressed as Equation (8):

(8) ∇(AB)=(A∇)B+(B∇)A+[AcurlB]+[BcurlA].

As shown in section 3.7.3, this formula leads to the derivation of Equation (1), based on Equation (5).

#### 3.1.1 Vector (Dot) Product

Abraham and Föppl refer to this type of vector product as the “inner” or “scalar” product [5, p.14], but this is now referred to as the vector “dot” product. The result is a number, called a scalar and the real part of Hamilton's quaternion. Whereas a scalar is a quantity entirely described by its magnitude (e.g., number, mass, temperature, speed), a vector has magnitude as well as direction. Let the point of action of a force **F** move with velocity **v**. The work done per unit time by this force is a scalar quantity given by the (dot) product of the magnitudes of the vectors **F** and **v** and the cosine of the angle between them (Equation 9):

(9) $$\mathrm{Fv}=\left|F\right|\cdot \left|v\right|\cdot \cos\left(F,v\right),$$

where |F| and |v| refer to the magnitudes of F and v. The cosine of the angle between **F** and **v** is equal to +1 when their directions are the same, −1 when they are in opposite directions, and 0 when they are normal (orthogonal—i.e., at right angles) to each other. The dot product of two vectors can therefore be defined in two‐dimensional space.

#### 3.1.2 Vector Cross‐Product

Abraham & Föppl refer to this type of vector product as the “outer” or “vector” product [5, p.17]. The term used now is “cross” product and for two vectors, **A** and **B**, results in a vector that can be defined only in three‐dimensional space as Equation (10):

(10) $$A\times B=\left|A\right|\left|B\right|\sin\left(A,B\right)n$$

where sin(A, B) refers to the sine of the angle between **A** and **B** in the plane containing them, and **n** is a unit vector perpendicular (normal) to this plane. The magnitude of this cross‐product is the area of the parallelogram that the vectors span. Conventionally, the so‐called “right‐hand rule” is employed to give the direction of **n**. The first finger (forefinger) points in the direction of **A**, and the middle finger in the direction of **B**, so that the thumb points in the direction of the unit vector **n**. Using this rule indicates that the cross‐product is not commutative—i.e., A×B=−(B×A). This is why, for Hamilton's quaternion algebra, ki = j, but ik = −j. Maxwell [17, pp. 25–31], however, employed the concept of a right‐handed screw for the cross product, which effectively reverses the direction of **n**. As a result, his definition and concept of field “convergence” are now referred to as field “divergence”.

### 3.2 Vector Fields

A scalar field is a mathematical function that connects a scalar quantity to each point in space. An important result of vector calculus is that a vector field is derived from the differentiation of its scaler field [5, p. 50]. This transforms the scalar field into a vector field that describes the rate and direction of change at each point of the scalar field. Let a scalar *ɸ* quantity vary so that it represents a continuous and differentiable function of location (*x, y, z*). In this scalar field, we position a small vector *d* **s** and consider the increment of *ɸ* as we change the location of *d* **s**. If *dx*, *dy*, and *dz* are the components of *d* **s**, and *d*s its length, the increment of *ɸ* is expressed as Equation (11):

(11) $$d\phi =\frac{\partial \phi }{\partial x}dx+\frac{\partial \phi }{\partial y}dy+\frac{\partial \phi }{\partial z}dz,$$

The rate of increase, we seek, is equal to the component of the vector ∂ϕ/∂x,∂ϕ/∂y,∂ϕ/∂z in the direction of *d* **s**. This vector **v** is called the “gradient” of the scalar *ɸ* at the point (*x*, *y*, *z*) and is written as Equation (12):

(12) $$v=\nabla \phi =i\frac{\partial \phi }{\partial x}+j\frac{\partial \phi }{\partial y}+k\frac{\partial \phi }{\partial z}.$$

A vector field **v**(*x, y, z*) has, therefore, been derived from the scalar field *ɸ*(*x, y, z*). The nabla symbol ∇ (also known as “del”) is Hamilton's gradient “operator” (Equation 13):

(13) $$\nabla =i\frac{\partial }{\partial x}+j\frac{\partial }{\partial y}+k\frac{\partial }{\partial z},$$

where ∇ is both a vector and an “operator” that acts on whatever follows (e.g., “addition” and “subtraction” signs are arithmetic operators). ∇ϕ implies an action on *ɸ* that differentiates it to give the gradient of the scalar field *ɸ*—which results in a vector field. Should the vector *d* **s** be directed along a surface where *ɸ* is constant, the scalar product of grad *ɸ* and *ds* is zero. The direction of grad *ɸ* thus coincides with the direction of the “steepest” increase of the scalar *ɸ*, with magnitude (Equation 14):

(14) $$\left|v\right|=\left|grad\;\phi \right|=\sqrt{{\left(\partial \phi /\partial x\right)}^{2}+{\left(\partial \phi /\partial y\right)}^{2}+{\left(\partial \phi /\partial z\right)}^{2}}.$$

As an example, on moving away from a point (*x*, *y*, *z*), the gradient of a scalar temperature field *T*(*x*, *y*, *z*) indicates the “direction” for which the temperature rises most quickly. The “magnitude” of the gradient indicates how “rapidly” the temperature increases along that direction. This informs us, with respect to Equations (4) and (5), that the ponderomotive (DEP) force drives a particle in the direction of the “steepest” gradient of the local field **E**.

Regarding Equation (7). For two paths, s and s', both of which lead from point 1 to 2 and are composed of individual line elements *d* **s** in the direction from 1 to 2, we can for each point on these curves form the scalar products Equation (15):

(15) $$\left(vds\right)=v_{s}ds=\left(\partial \phi /\partial s\right)ds,$$

where **v** is the vector field. Summing for every *ds*, and going to the limit *d* **s** → 0, the following “line” integrals are derived (Equations (16), (17)):

(16) $$\oint_{1}^{2}v_{s}ds=\oint_{1}^{2}\left(\partial \phi /\partial s\right)ds=\phi_{2}-\phi_{1},$$

(17) $$\oint_{1}^{2}v_{s}d\;s^{\prime }=\oint_{1}^{2}\left(\partial \phi /\partial s^{\prime }\right)\;d\;s^{\prime }=\phi_{2}\;-\phi_{1}.$$

These line integrals have the same value for any two paths that connect two definite points of the field. They go to zero for any closed path that starts and ends at the same point *p*, so that (Equation 18):

(18) $$\oint_{p}^{p}v_{s}ds=0.$$

If the vector field **v** *_s_* is a force field, then (−*ɸ*) is called the scalar “potential”. If the total work done by a force to move a particle between two points is independent of the path taken, this force is defined as a “conservative” force. The work done depends only on the initial and final position of the particle. Examples of a conservative force include the gravitational force and the electric force. No useful work can be achieved by carrying a 1 kg mass repeatedly up and down the same hill, no matter the paths taken. Work is done (by something) to oppose the gravitational field in moving a stationary mass to the top of the hill, whereas an equal but opposite amount of work is done by the field in moving the mass back down to rest at its initial place. The work–energy theorem, which states that the work done by the sum of all forces acting on a particle equals the change in the kinetic energy of the particle, has also been conserved. This comparison with the gravitational force was used in the development of electric field theory to define −*ɸ* as the scalar electrical “potential”.

**FIGURE 3:**
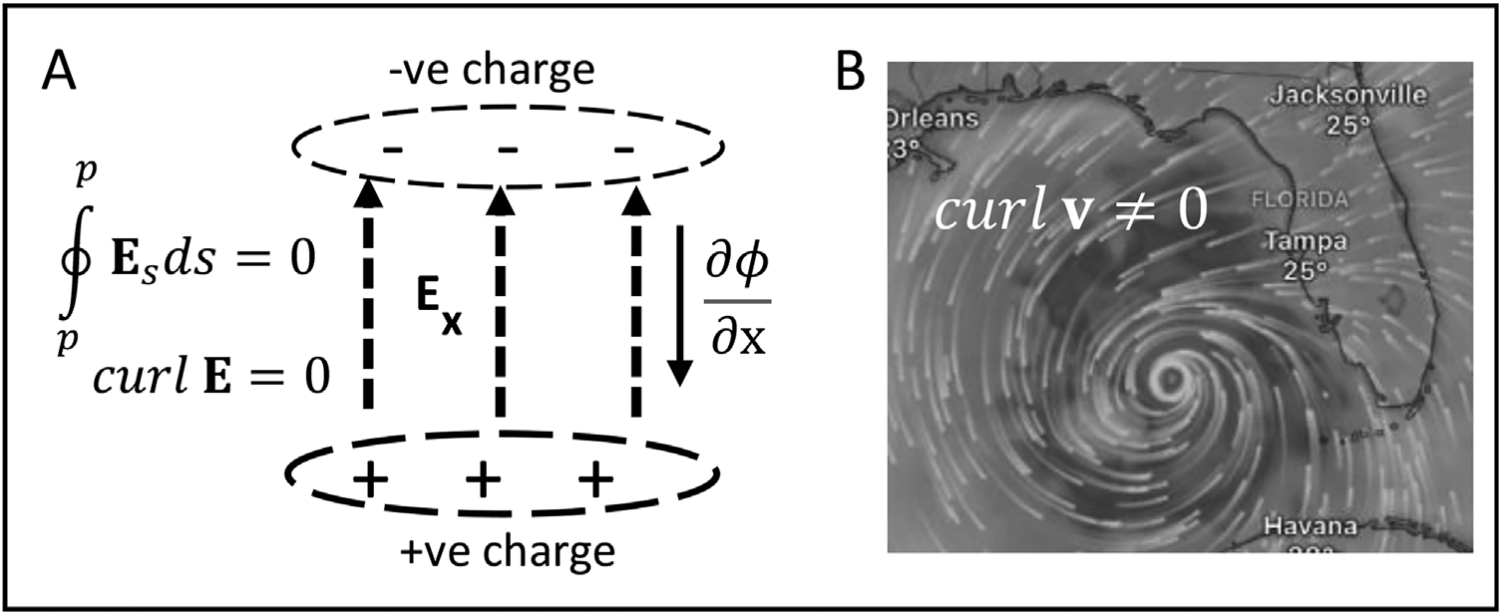
A, The electric field, defined as the negative gradient of the electric potential *ɸ*, is curl‐free (i.e., curl E = 0). B, The field of wind speeds near Florida on October 9, 2024, was not curl‐free (Windy.com).

Abraham & Föppl classify the special case of a vector field that can be expressed as the gradient of a potential (v=−∇ϕ, or v=−gradϕ), as an “irrotational” vector field (“wirbelfrei”—vortex‐free). This is illustrated in Figure 3 and stated as the theorem:

- The field of the gradient of a scalar ɸ is always an irrotational field [5, p. 52].

The converse of this theorem is also true:

- An irrotational vector field can always be regarded as the field of the gradient of a scalar [5, p. 53].

The rotation of a vector **v** is designated as “*curl* **v**”, and to be irrotational implies that *curl* **v** = 0. The irrotational, curl‐free, property of the electric field is illustrated in Figure 3A. Electric lines of force are shown, following accepted convention, directed from a positive charge and terminating at an equal but negative charge. The strength of the electric field E is indicated by the spacing between the lines of force. An example of a field that is not curl‐free (wirbelfrei) is shown in Figure 3B. According to Maxwell's Equation describing Faraday's Law of induction, an electric field that is not curl‐free is produced by a conductor carrying a time‐varying current. This is discussed further in sections 3.4.1 and 4.5.2.

The dot product of the grad operator ∇ and a vector field **v** gives the divergence of that field (div **v**) expressed as Equation (19) [5, p.55]:

(19) $$\mathrm{div}\;v=\nabla \cdot v=\frac{\partial v_{x}}{\partial x}+\frac{\partial v_{y}}{\partial y}+\frac{\partial v_{z}}{\partial z}.$$

If div **v** is required in the direction of the positive (i.e., outward), normal, direction at a surface, this differentiation is signified as ∂/∂n.

#### 3.2.1 Divergence Theorem

The surface integral of a vector field over a closed surface is called the “flux” of that vector field. The divergence theorem states that this surface integral is equal to the volume integral of the divergence of the field over the region that is totally enclosed by this surface. This is expressed as Equation (20) [5, p.57]:

(20)

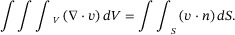




The left‐hand side of this equation gives the volume integral of the field divergence, with the right side giving the integral of the field over the enclosing surface. The vector **n** is the unit normal vector pointing outwards from each unit area of the surface. Equation (20) basically states that the algebraic sum of the enclosed sources and sinks of the field produces the net flux flowing out of (or into) that region.

#### 3.2.2 Laplace's Operator

Maxwell expresses the opinion that one of the most remarkable properties of the grad operator ∇ is that when repeated it becomes Equation (21):

(21) $$\nabla \cdot \nabla =\nabla^{2}=\frac{\partial^{2}}{\partial x^{2}}+\frac{\partial^{2}}{\partial y^{2}}+\frac{\partial^{2}}{\partial z^{2}},$$

and is “an operator appearing in all parts of Physics” [17, p. 31]. It is used for describing electric and gravitational potentials and applied in differential equations of molecular diffusion, heat flow, fluid flow, image processing, and computer vision. An example of its use is given in section 3.6.4.

### 3.3 Field Intensity and Flux

The important feature of Maxwell's theory of the electric field is that a field's intensity is associated with every point in space. Both the magnitude and direction of the field can be defined and so is treated as a vector field **E**, whose physical significance at a specific point in space only emerges if a small “testing body” carrying an electric charge *q*_1_ is brought up to that location. A vector force **F** acts on this charged body given by Equation (22):

(22) $$F=q_{1}E$$

There are restrictions on the validity of this equation. It ceases to hold exactly if the ‘probe body’ *q*_1_ is too close to the electrified body (e.g., the fixed sphere in Figure 2) that creates **E** and if this field varies too rapidly with the change of location. As investigated by Boltzmann [3] these restrictions are minimized if the physical size of the probe body is sufficiently small, and if it carries a sufficiently weak charge that is far enough away to not influence the surface charge distribution on the fixed sphere in Figure 2. A measurable force appears only when a second charge *q*_2_ is brought up to the neighborhood of *q*_1_. The magnitude of the force **F** that each of these two charges exert on each other when at rest in free space (vacuum), at a distance *r* apart, is given by Coulomb's Law Equation (23):

(23) $$\left|F\right|=k\left[q_{1}q_{2}/r^{2}\right].$$

The direction of the force is along the line joining the charge centers. The parameter *k* is a constant whose numerical value depends on the units of measurement. Boltzmann, Föppl, and Hatfield used the absolute electrostatic system of units (the CGS‐ESU system). In this system, two equal point charges spaced 1 cm apart in free space are of one electrostatic unit (esu) each if the electrostatic force exerted on each other is 1 dyne. Thus, *k* = 1 in Equation (23). For the international SI system, the unit of force is one newton (N), the unit of charge is one coulomb (C) and length has the unit of one meter (m). In this case, the value of *k* in Equation (23) is given as $k=1/4\pi \varepsilon_{o}$, where *ε*_o_ is the electric constant (also known as the absolute permittivity of free space) of magnitude 8.854 x 10^−12^ C^2^ N^−1^ m^−2^. *k* thus has a value close to 9 x 10^9^ N m^2^ C^2^. From Equations (23) and (24) the result of Coulomb's experiments can be described by stating that charge *q*_1_ generates an electric field given by:

(24) $$E=q_{1}/r^{2}\left(\mathrm{CGS}-\mathrm{ESU}\;\mathrm{system}\right).$$

The field radiates out from the point source with spherical symmetry and decreases in magnitude as an inverse square of radial distance. This is where Maxwell's theory departs from that of “action at a distance”. Abraham & Föppl [8, p.131] explain that electric charge is both the source of the flow of electric force and the point of attack of the electric force. In other words, electric charges are no longer considered as centers of force, but as sources of electric force. This is emphasized by them in using the terms “Quellpunkte” (meaning point source) and “Doppelquelle” to describe a dipole.

### 3.4 Gauss's Law and Boltzmann's Nonuniform Field

Gauss's Law states that the total electric flux **Φ** *_E_* through any closed surface that surrounds a defined volume is proportional to the net charge *Q* located within that volume. It does not matter how the constituent charges are distributed, so long as they are all located within this volume. This is described by Equation (25):

(25)

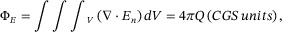

for any volume containing a net charge *Q*, and where **E** *_n_* is the field crossing normal to its boundary surface. According to the divergence theorem (Equation 20) this is equivalent to Equations (26):

(26) $$\int \int_{S}\left(E_{n}\cdot dA\right)=4\pi p\left(CGS\;units\right),$$

where *ρ* is the volume charge density. Based on Equations (25) and (26) we can write (Equation 27):

(27) $$divE=\nabla \cdot E_{n}=4\pi \rho \left(CGS\;units\right).$$

$$\nabla \cdot E_{n}=Q/\varepsilon_{o}\left(SI\;units\right)$$

Gauss's law can be used in two ways. The spatial distribution of source charges can be determined by measuring the electrical field everywhere around them, or with knowledge of the charge distribution the generated electric field can be determined. The latter application is useful for applications of DEP when we wish to determine the electric field at a given point produced by conductors of known electric potential. This given point must lie on the surface of the Gaussian sphere. Because Equations (20) and (25) do not specify the size of the enclosed volume, it can be a sphere of any radius—so long as all the charges are contained within it.

#### 3.4.1 Maxwell's Equations

In his unified theory of electromagnetism, Maxwell developed equations that couple electric and magnetic fields. Two of these four equations (also known as the Maxwell‐Heaviside Equations) apply to the electric field, whilst the magnetic field is described by the other two equations. Equation (27) (Gauss's Law) is a twin of Maxwell's Equation known as Gauss's Law for magnetism (∇·B=0). The other equation describing the electric field is based on Faraday's Law of induction (Equation 28):

(28) $$\nabla \times E=-\frac{1}{c}\frac{\partial B}{\partial t}\left(\mathrm{CGS}\;\mathrm{units}\right),$$

where **B** is the magnetic field and *c* the speed of light. In SI units this is given as Equation (29):

(29) $$\nabla \times E=-\;\frac{\partial B}{\partial t}=-\mu_{o}\frac{\partial H}{\partial t}\;\left(\mathrm{SI}\;\mathrm{units}\right),$$

where **H** is the magnetizing field, $\mu_{o}$ is the magnetic constant (permeability of free space), and *c* is given by $c={\left(\varepsilon_{o}\mu_{o}\right)}^{-1/2}.$ These equations show that if the E‐field in a capacitor is changing with time (e.g., as Cos *ωt*) it will induce a circular H‐field that in turn induces a counter E‐field. Abraham & Föppl's classification of an irrotational vector field, characterized by the property ∇×E=0, is therefore only strictly applicable to an electrostatic field. However, as discussed in section 3.6.3, all the equations presented in this article so far can be considered to satisfy quasielectrostatics conditions, provided that the frequency of the electric field is below 500 MHz. In this case, the factor −∂B/∂t in Equations (28) and (29) can be neglected and so no correction is required of the electric field magnitude.

#### 3.4.2 The Nonuniform Field in Boltzmann's Experiments

**FIGURE 4:**
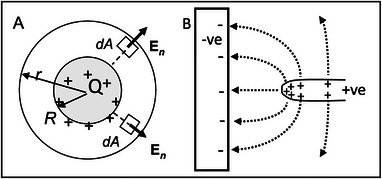
Spherical Gaussian surface drawn around a fixed sphere of Figure 1. The field divergence is equal to 4π*Q*. B, Lines of electric force originating from a pin electrode acting as an anode and terminating at a flat plate cathode.

In Figure 2, a total charge 2*Q* is assumed to have been transferred to the charging terminal from the influence machine. If the surface areas of the connecting wires are relatively small, this charge is assumed to be equally divided and distributed over the surfaces of the two fixed metal spheres having the same radius. Let *R* be this radius and, as shown in Figure 4A, construct an imaginary Gaussian sphere of radius *r* around one of them.

For every infinitesimal surface area *dA* of this Gaussian sphere (total surface area 4*πr* ^2^) the field **E** *_n_* crossing normal to its surface is the same, so that (Equation 30):

(30) $$\int \int_{S}\left(E_{n}\cdot dA\right)=4\pi r^{2}E.$$

Thus, from Equation (26):

(31) $$E=kQ\;1/r^{2}\;\left(r\;>\;R\right).$$

Inside the metal sphere the field is zero, so this equation (with *k* = 1 in CGS‐ESU units) gives the field intensity at a radial distance *r* from its center and beyond its radius *R*. The dependence of **E** on the total surface charge *Q* explains Ettinghausen's required assistance checking its relative magnitude for each experiment.

The test and reference spheres shown in Figure 2 experience nonuniform fields given by Equation (31). Gaussian spheres constructed around them would contain no charge—their field divergence is zero. The term divergent electric fields used by Pohl [4] should more correctly have been written as nonuniform electric fields.

### 3.5 The Electrostatic Potential

As depicted in Figure 3, because the electric field is irrotational it can be equated to the negative gradient of a scalar potential *ɸ*

E=−∇ϕ,

the electrostatic potential at a location *r* is then identified as *ɸ*, given by Equation (32):

(32) $$\phi =q_{1}/r.$$

The x‐components of the electric field are given by Equation (33):

(33) $$E_{x}=-\partial \phi /\partial x=\left(q_{1}/r^{2}\right)\cdot \left(x/r\right)=\left(q_{1}/r^{2}\right)\cos\left(r,x\right),$$

with similar equations for *E*_y_ and *E*_z_. Based on Theorems 1 & 2, this confirms the depiction given in Figure 3 of the electric field as an irrotational (curl‐free) vector. The decrease of the electrostatic potential from point 1 to 2 is equal to the line integral of **E** taken over any path s between these points:

$$\phi_{1}-\phi_{2}=\oint_{1}^{2}Eds$$

If the source of the field **E** consists of a number *n* of point charges, their fields are superimposed, and the resultant electrostatic potential is given by Equation (34):

(34) $$\phi =\sum_{i=1}^{n}\left(q_{i}/r_{i}\right).$$

#### 3.5.1 Definition of Electrostatic Potential and Potential Energy

Place a unit of positive charge at a location *r* that is an infinite distance away from the influence of any other charge. This is defined as the reference zero of potential. The potential ϕ at a given point is equal to the work that must be done by an external agent to bring that unit of positive charge from an infinite distance (or from any place where the potential is zero) to that point [17, p. 78]. Two charges of the same sign repel each other, and so work must be done by an external agent to bring them together from infinity. The amount of work done is the potential energy of this couplet of charges. Now consider two distributions of charges, each occupying small volumes and of magnitudes *q*_1_ and *q*_2_, consisting of individual charges each brought from infinity and placed distance *r*_12_ apart. If this separation distance of *r*_12_ is large compared with the spatial extent of the charge distributions *q*_1_ and *q*_2_, the work *W*_2_ required to assemble them is their mutual potential energy given by Equation (35):

(35) $$W_{2}=q_{1}q_{2}/r_{12}.$$

Repeating this procedure to construct an assembly of *N* charges *q*_1_, *q*_2_, *q*_3, .…_ *q*_N_, of mutual separation distances *r*_12_ … *r*_13 …_ *r*_N‐1, N_ requires the total work *W_N_*, calculated as the sum of all possible mutual pair energies given by Equation (35):

(36) $$W_{N}=1/2\left(\sum_{i=1}^{N}\sum_{j=1}^{N}\left[q_{i}q_{j}/r_{ij}\right]\right).$$

Expressing the summations in this way ensures that any terms involving *Ii* = *j* are not included, so avoiding any pair *ij* being counted twice. This explains the presence of the factor ½ in Equation (36). Because work *W_N_* is being done on the field by an external agent to bring charges of the same polarity together then, based on the Work‐Energy theorem, there will be an increase in the potential energy of the system of charges, given by $\Delta U=W_{N}$. Equation (36) thus represents the electrical energy of the system expressed in terms of the distribution of the charges at different parts of the system and their potentials [17, vol 1, p. 104].

#### 3.5.2 Poisson's and Laplace's Equations

These are important, so‐called elliptic, partial differential equations [23, pp. 962‐971] of wide application. For Poisson's equation (Equation 37):

(37) $$\nabla^{2}u=u_{xx}+u_{yy}+u_{zz}=f\left(x,y,z\right).$$

With function *f* already known, the objective is usually to determine the real or complex‐valued function *u*. From Equations (28) and (31), Equation (38) can be expressed as:

(38) $$\nabla \cdot E=\nabla \cdot \left(-\nabla \phi \right)=-\nabla^{2}\phi =\rho ,$$

to produce Poisson's Equation for electrostatics as Equation (39):

(39) $$\nabla^{2}\phi =-\rho ,$$

where *ρ* is the charge density per unit volume contained in the region of interest.

Laplace's equation is a special case of Poisson's equation Equation (40):

(40) $$\nabla^{2}u=u_{xx}+u_{yy}+u_{zz}=0,$$

to give a solution of *φ*, for a volume containing no charges, in terms of the Laplacian operator (Equation 21) (Equation 41):

(41) $$\nabla^{2}\phi =\frac{\partial^{2}\phi }{\partial x^{2}}+\frac{\partial^{2}\phi }{\partial y^{2}}+\frac{\partial^{2}\phi }{\partial z^{2}}=0.$$

### 3.6 The Distribution of Electricity on Conductors

The term “conductor” implies a metal. Their atomic structures consist of positively charged atomic nuclei surrounded by negatively charged electrons. The net positive charge of the atomic structure is exactly balanced by that of the electrons. Roughly speaking, one electron per atom is delocalized and free to move in the atomic structure. The term “sea of electrons” is sometimes used to describe this situation. On exposure to an electric field and to achieve electrical equilibrium, they redistribute themselves quickly within an atomically thin layer at the surface of the conductor to produce a counterfield. At equilibrium, this redistribution of charge ceases. The state is quickly reached and everywhere inside the metal has the same potential. This is expressed as the theorem:

- The electrostatic potential φ is constant in the interior of a conductor [8, p.133].

Based on the definition E=−∇ϕ, this also means that conductors cannot support an electric field. Therefore, no component of an external electric field can exist parallel to the surface of a conductor—external lines of electric force only meet its surface at right angles. When a conductor's surface is shown as positively charged, this does not indicate that free positive charges (e.g., positrons) are at this surface, but that the number density of electrons within the atomically thin layer at the surface is insufficient to balance the positive charges of the atomic nuclei. The excess electrons have redistributed themselves elsewhere on the surface. This superficial charge distribution, of charge density *ω* per unit area, produces an outward pointing field *E* **_n_**, where **n** signifies it is normal to the surface, given by Equation (42):

(42) $$E_{n}=4\pi \omega =-\partial \phi /\partial n,$$

#### 3.6.1 Thomson's Theorem

When charges are induced to move in an electric field, the energy of the field is reduced by the amount of work done. The charges of electrification distribute themselves on a conductor's (or dielectric's) surface in a way that the field energy becomes a minimum. This is known as Thomson's theorem [8, p. 172] and is presented as a footnote in his editing of the 3rd edition of Maxwell's Treatise [17, p. 154]. Over a flat surface of a conductor, the charges of electrification are uniformly distributed, and external lines of electric force that start or end at them are parallel to signify that the field is uniform. However, for a surface that curves the charges repel each other less strongly and pack more closely in inverse proportion to the radius of curvature. This is depicted in Figure 4B where the surface charges are closer together at the tip of a pin electrode so that the lines of force converge to produce a local field maximum and field gradient. The lines of force that terminate at the flat surface of the cathodic electrode are spaced farther apart and tend toward being parallel so that the field intensity there is weaker and more uniform than at the pin electrode. The DEP force acting on a particle will thus be greater near the pin electrode than near the plate electrode. Hatfield took this into account in his design of electrodes [1, 6].

#### 3.6.2 The Fundamental Problem of Electrostatics

As stated by Abraham & Föppl [8, p.135] this problem is appreciated by first noting that in space free from charge the electrostatic potential *φ* is given by Laplace's equation as Equation (43):

(43) $$div\;E=-div\;grad\;\phi =-\nabla^{2}\phi =0.$$

In principle then, if the field **E** is known, the distribution of charge density *ω* on the electrode surface can be found from Equation (42) ($E_{n}=-\partial \phi /\partial n$). Alternatively, if ω is known then **E** can be calculated from Equation (32) (ϕ=Q/r). For each separate conductor we know either its potential *ɸ* or its total charge *Q*. We do not know both values. The relationship between *ɸ* and *Q* is given by Equation (44):

(44) $$Q=\int \omega dS=-\frac{1}{4\pi }\int \left(\partial \phi /\partial n\right)dS.$$

We are required to find the corresponding solution of Laplace's equation. However, the problem we face is that *ω* is the equilibrium charge density defined by the electrostatic field **E**. Instead, we require knowledge of the potential *ɸ* for just one of the electrodes, because then *ɸ* can be found everywhere else and with its local gradient the value of **E**.

#### 3.6.3 Quasielectrostatics

At the 2024 DEP conference in Dublin, several presentations described the development of DEP and related electrokinetic experiments at frequencies up to 500 MHz and potentially higher. To what extent can the factor −∂B/∂t in Equation (28) be neglected at such high frequencies? Are errors introduced in the analysis of DEP experiments if energy losses due to electromagnetic (em) radiation are neglected, for example? Stratton [24, p.438] analyzed the dependence of the energy of em radiation on its wavelength and concluded that so long as this is long compared with the largest dimension of the metallic circuit, em radiation loss is negligible. The wavelength *λ* is given by *λ* = *c*/*f* where *f* is the em frequency. At 1 GHz the wavelength is 30 cm, and so for most DEP systems the factor $-\frac{1}{c}\frac{\partial B}{\partial t}$ in Equation (28) is not significant.

The theory of the electric field and its forces described so far here assumes that they are operating in free space. As taught by Abraham & Föppl, there are two classes of material body. One class, like a metal wire, can communicate electricity from the pole of a battery to another body. Bodies that have this property are called “conductors of electricity”, those that lack it are called insulators. In pure electrostatics, there are only conductors and insulators (i.e., ideal dielectrics) and no other classes in between. The classical theories of quasi‐electrostatics and electrostatics are thus the same. However, these two classes of material are not always strictly separable. For example, there are bodies, such as the Nernst incandescent body, which at ordinary temperatures are insulators, but become conductors when heated [8, p. 58]. The dielectric media between electrodes in DEP experiments are rarely ideal insulators—they support ionic currents, for example. In this case, the equations describing the electrical polarization of solid bodies should consider electrical conduction losses and the fact that the polarization displacement current may not be in step (phase) with the applied electric field. This is considered in section 4.5.2.

#### 3.6.4 Simple Application of Laplace's Equation

As shown in Equation (32), the quantity of charge *Q* at equilibrium on the surface of a conductor and the associated potential *ɸ* are directly related. The quotient of these two quantities depends only on the geometrical shape of the conductor and the position of any conductors adjacent to it. This quotient, *Q*/*ɸ* is called the self‐capacity *C_s_* of the single conductor (Equation 45):

(45) $$C_{s}=Q/\phi .$$

Consider two conductors *A* and *B* that take the form of equally large, flat plates, located at a constant distance *d* from each other. This arrangement was originally called a compression apparatus for electricity or a condenser [5, p.262]. It is now called a capacitor. We wish to find the capacitance of such a geometrical arrangement.

If the distance *d* is very small compared with the surface area *S* of the plates, then apart from at their edges, the potential *ɸ* only varies in the direction perpendicular to both plates. If this is chosen for the *z*‐axis, *ɸ* becomes independent of *x* and *y*, and Equation (41) reduces to Equation (46):

(46) $$\nabla^{2}\phi =\frac{\partial^{2}\phi }{\partial z^{2}}=0.$$

A function of *ɸ* that satisfies this equation is

ϕ=az+b.

Let the origin of the system of coordinates be on plate *B*, which is connected to the earth's potential as a reference zero potential. Thus *b* = 0 because *ɸ* = 0 at z = 0. From Equation (42), we now have Equation (47):

(47) $$a=\frac{\partial \phi }{\partial z}=-\frac{\partial \phi }{\partial n}=4\pi \omega =4\pi Q/S.$$

At plate *1*, z = *d*, so that ϕ=4πQd/S. Based on the definition of capacitance *C* given by Equation (45) as the ratio *Q*/*ɸ*, then (Equation 48):

(48) $$C=\left(S/4\pi d\right)\;\left(\mathrm{CGS}\;\mathrm{units}\right).$$

The value for *C* can become very large if the separation distance between plates A and B is made very small. Hence the original name “condenser”.

Faraday found that the charge storage (capacity) of a condenser depended on the type of insulating material (dielectric) that was inserted between its parallel plates. The capacity *C* was always larger when the dielectric was glass, sulfur, or turpentine oil, for example, instead of air. It did not matter if a small gap existed between the surfaces of the plates and the dielectric, provided that the width of this gap was small compared with the distance between the plates. The factor *ε* by which *C* was multiplied was found to be a constant that characterized the dielectric used. This constant was thus named the “specific inductive capacity” by Faraday—a term later changed to “dielectric constant” [8, p. 144]. This is compatible with Drude's description [5, p.263) of the fact that if two electrified small metal balls, which contain the charges *q*1 and *q*2, are placed in a well‐insulating liquid, for example, turpentine oil, they exert a smaller force on each other than if they were placed in the air or a vacuum. When placed in a vacuum, the force is expressed as Equation (49):

(49) $$K_{\mathrm{air}}=e_{1}e_{2}/r^{2},$$

whilst that taking place in turpentine oil, for example, is expressed as Equation (50):

(50) $$K_{\mathrm{oil}}=e_{1}e_{2}/\varepsilon r^{2},$$

where the factor *ε* must be greater than the value of 1 for air (*ε* is about 2.2 for turpentine oil). The factor *ε* is the dielectric constant. Equation (48) can thus be modified to allow for a dielectric other than air between the electrode plates (Equation 51):

(51) $$C=\left(S\varepsilon /4\pi d\right)\left(\mathrm{CGS}\;\mathrm{units}\right)\;,$$

or $C=(S\varepsilon \varepsilon_{o}/d)$(SI units), where *ε* is the dielectric constant of the material between the two plates, each of surface area *S*.

#### 3.6.5 Potential Energy Stored in a Charged Capacitor

Based on Equations (36) and (45), the increment of work *dW* required to add an elemental charge *dq* to a capacitor is expressed as Equation (52):

(52) $$dW=vdq=\frac{qdq}{C}.$$

The total work *W* required to fully charge a capacitor is given by integrating these increments (Equation 53):

(53) $$W=\int_{0}^{W}dW=\int_{0}^{Q}\frac{1}{C}qdq=Q^{2}/2C.$$

Based on the work–energy theorem equation, the potential energy *U* of the charged capacitor can be expressed as Equation (54):

(54) $$U=Q^{2}/2C=\frac{1}{2}C{\left(\phi_{A}-\phi_{B}\right)}^{2}.$$

The factor $\left(\phi_{A}-\phi_{B}\right)$ is the potential difference applied across the plates of the capacitor when fully charged, and is commonly written as V.

### 3.7 Induced Polarization of Spherical Bodies

Boltzmann [2] was absorbed with the fact that an electric field exerts a force on a metal or insulating body even though it may not itself be electrified. This force could only be by virtue of it being in a state of induced electrostatic polarization—even if it should be connected to the earth's potential. His experiments included the simultaneous scrutiny of the ponderomotive force acting on a small metal sphere connected to the earth's potential (as a fixed reference) and a small metal sphere insulated from sources of electrification. These cases of induced polarization are not described by Abraham & Föppl. However, by citing the method of images devised by Thomson [25] they describe the concept of electrostatic induction by considering the case of a point charge placed in front of a conducting plane of infinite extent [8, pp. 103–106].

#### 3.7.1 Method of Images: Charged Body near a Conducting Surface

**FIGURE 5:**
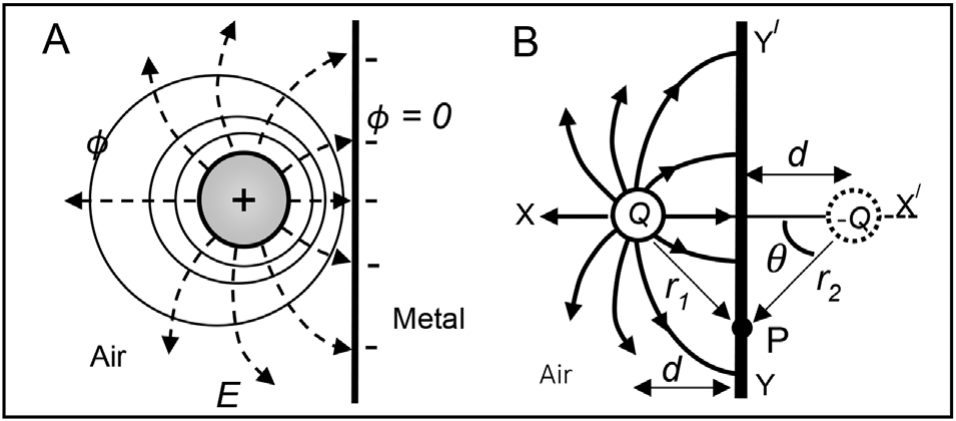
A, An electrified body located close to a metal surface. B, The charge *Q*, together with its image charge −*Q*, produces a field that satisfies the requirement that the potential is constant along the metal surface YY^/^.

Figure 5 shows how the potential contours and field lines around a charged body are distorted if it is placed close to the surface of a metal. An electric field cannot exist inside or along the surface of this metal and so, from the viewpoint of this charged body, the field it produces can no longer be derived using Equations (32) and (34).

In principle, as for the example given in section 3.6.4, the potential at all points outside the metal's surface can be obtained by solving Laplace's equation with the boundary condition that YY^/^ is a surface where the potential is zero. However, the technique known as the method of images provides a simple way to avoid this calculation. It also serves to introduce the concept of electrostatic induction and can be extended to consider the polarization of small metal spheres.

In Figure 5B, the distribution of charges along YY^/^ that satisfies Laplace's equation can be obtained by placing an imaginary charge −*Q* on line XX^/^ at a distance *d* behind the metal surface YY^/^. The lines of flux that converge on this “mirror” charge exactly mirror those that originate from the original charge *Q* and meet surface YY^/^ at right angles to it. This can be shown by considering the potential at any radial location *P* outside the metal boundary, where (Equation 55):

(55) $$\phi =\phi_{1}+\phi_{2}=Q/r_{1}-Q/r_{2},$$

The value of *ϕ* is zero wherever $r_{1}=r_{2}$. This occurs at all points where *P* is located along the conductor surface YY^/^. The field normal to this conducting surface is (Equation 56):

(56) $$E_{n}=-\frac{\partial \phi }{\partial n}=-Q\frac{\partial \left(\frac{1}{r_{1}}\right)}{\partial n}-Q\frac{\partial \left(\frac{1}{r_{2}}\right)}{\partial n}=-\frac{2Q}{r^{2}}cos\theta =2Qd/r^{3},$$

corresponding to a distribution of surface charge (Equation 57):

(57) $$\omega =\frac{1}{4\pi }E_{n}=-Qd/2\pi r^{3},$$

The induced charges on the metal's surface are therefore distributed in such a way that their surface density is inversely proportional to the cube of the distance from the real point charge. The potential *ϕ*_2_ of this induced distribution of charges on the metal surface at any point outside the metal is the same as would be produced by the image charge −*Q* acting alone. Likewise, the calculation of the sum of the forces of attraction between charge *Q* and the induced negative charges distributed on the conducting surface is reduced to the simple calculation using Coulombs Law:

$$F=Q^{2}/\left(2d^{2}\right)$$

#### 3.7.2 Method of Images: Metal Sphere in a Uniform Field

**FIGURE 6:**
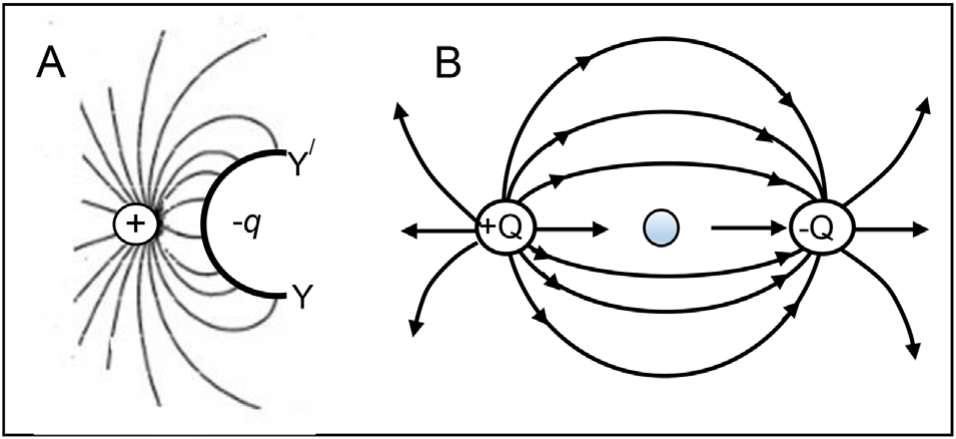
An approach to understanding the polarization of a metal sphere. A, Change the metal surface YY^/^ of Figure 5A into a semicircle and determine the image charge −*q*. B, Place charges +*Q* and −*Q* each side of the sphere.

Finding the distribution of induced charges on the surface of a metal sphere or ellipsoid in either the field of a point charge or in a uniform field is a standard problem taught in classical texts on electrostatics or electromagnetic theory. It involves solving Laplace's equation using a method known as the separation of variables to satisfy the given boundary conditions. Bessel or other forms of harmonic function, together with associated Legendre equations, are employed (e.g., [24], pp. 201‐211). It can be said that many students find this to be an acquired rather than an innate taste. It is avoided by Abraham & Föppl. Extending the method of images, as outlined in Figure 6, is suitable for our purposes here.

The concept to be adopted is outlined in Figure 6. The field lines of charge *Q* can pass around the metal surface YY^/^ after it is bent into a semicircle. An analysis is then made of the factors that control the magnitude and location of the image charge −*q*. To simulate the situation of a small metal sphere in a uniform field, add an external charge −*Q* as shown in Figure 6.B. This creates an added image charge of +*q* inside the sphere to form, with −*q*, a dipole. As the magnitudes of ±*Q* and their distance apart are increased, the field lines in the vicinity of the small metal sphere become more parallel. However, to achieve this analysis, a template must first be found to establish a spherical surface of zero potential. This is described in Appendix 1.

As shown in Appendices 2 and 3, the method of images can also be applied to the cases of a point charge *Q* influencing either a metal sphere connected to the earth's potential, or a sphere insulated from external electrification. For the case of an insulated and previously uncharged metal sphere, an additional image charge +*q* must be added inside the sphere to neutralize the image charge −*q* previously placed at the sphere's center for the case of an earthed sphere. As *Q* is moved away from the sphere's surface the image charge *+q* moves toward the center of the sphere, so that with −*q*, forms a point dipole moment. The field *E*_o_ produced by *Q* also becomes more uniform in the vicinity of the sphere. For the scheme depicted in Figure 6B, it is shown in Appendix 4 that:

- A conducting sphere placed in a uniform electric field **E**_o_ is polarized in such a way that the induced charges are distributed on its surface to produce an external field equivalent to that of a point dipole moment **m** located at the sphere's center.

The magnitude of the induced dipole moment **m** is given by (Equation 58):

(58) $$m=E_{o}\cdot R^{3},$$

where *R* is the radius of the sphere. The induced moment is aligned with the direction of the external uniform field E_o_. This result agrees with that obtained by Stratton from a formal solution of Laplace's equation involving spherical harmonics and the associated Legendre function [25, pp. 201‐205].

#### 3.7.3 Ponderomotive (DEP) Force Acting on a Metal Sphere

Based on Equations (5) and (58), together with Boltzmann's stipulation [5, p. 49] the sphere is small enough so that the flow of the electric lines of force within it is almost the same as if it were in a uniform field **E**_o_, the ponderomotive (DEP) force acting on a polarized metal sphere is given by Equation (59):

(59) $$F_{\mathrm{DEP}}=m\cdot \frac{\partial E}{\partial x}=\left(m\cdot \nabla \right)E_{o}=R^{3}\left[E_{o}\cdot \nabla \right]E_{o},$$

Furthermore, based on Equations (4) and (14), the metal sphere is driven by this force in the direction of the steepest gradient of the local field toward a greater field strength. Now is an appropriate time to consider Equation (8):

$$\nabla \;\left(AB\right)=\left(A\nabla \right)\;B+\left(B\nabla \right)A+\left[A\;\mathrm{curl}\;B\right]+\left[B\;\mathrm{curl}\;A\right]$$

Identifying that in our case $A=B=E_{o}$, this equation can be written as Equation (60):

(60) $$\nabla E_{o}^{2}=2\left(E_{o}\cdot \nabla \right)E_{o}+2\left[E_{o}\mathrm{curl}E_{o}\right].$$

The field is “wirbelfrei”, so curl **E_o_** is zero. An alternative to Equation (59) is thus expressed as Equation (61):

(61) $$F_{DEP}=\frac{1}{2}R^{3}\nabla E_{o}^{2}$$

Based on the work–energy theorem, encapsulated by Equation (7), the energy which must be spent to remove a metallic sphere of radius *R* from a field E, into a region where there is no field, is equal to $1/2(R^{3}E^{2})$. The **E** ^2^ dependence shown by Equation (61) also indicates that the direction of **F** *_DEP_*, with or against the field direction, does not depend on the direction of the field and explains why Boltzmann could perform a ponderomotive force measurement with an alternating field (∼100 Hz). To appreciate the full extent of Boltzmann's investigations requires an understanding of the induced polarization of a dielectric sphere.

## 4 Dielectrics

This subject comprises many mathematical, physics, and experimental facets. Only those of direct relevance to the following two rules claimed by Hatfield in his patent of 1924 [1] are appraised here:

- If insulating particles are replaced by conducting ones, they behave as if their dielectric constant were very large and always move to the strongest part of the field.
- The rule is that the particles move to increase the dielectric capacity of the system.

The first rule is embraced by Boltzmann in his exploitation of the fact that “a small sphere of a dielectric material having a dielectric constant ε, that is electrified only by induction, will experience a force in free space some [(ε−1)/(ε+2)] times that of a small metal sphere of equal size and similarly electrified only by induction”. The second part of the first rule has been addressed in section 3.7.3. We now consider the particulars of why conducting (i.e., metal) particles behave as if their dielectric constant were very large.

### 4.1 Conductors Exhibit Very Large Dielectric Constants

**FIGURE 7:**
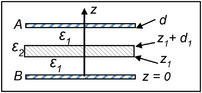
Two slabs of dielectric material, *ε*_1_ and *ε*_2_, between the metal plates *A* and *B* of a capacitor. (Based on Drude [5, p.268]).

There are several ways to validate this section's title and Drude [5, p.268] offers the simplest by applying the capacitor geometry analyzed in section 3.6.3 with Equation (51). The capacitor geometry is shown in Figure 7.

As shown in Figure 7 the overall capacitor of capacitance *C* has a thickness *dd_1_* and consists of a dielectric slab of thickness *d_1_* and dielectric constant *ε*_2_ located within a medium of dielectric constant *ε*_1_ and total thickness *d_1_*. The two dielectrics have the same surface charge, and the potential difference between their series combination is equal to the sum of their surface potential differences. It follows from Equation (45) that the reciprocal value *C* is equal to the sum of the reciprocal values for *C_1_* and *C_2_* (Equation 62):

(62) $$1/C=1/C_{1}+1/C_{2}=4\pi /S\left[\left(d-d_{1}/\varepsilon_{1}\right)+\left(d_{1}/\varepsilon_{2}\right)\right].$$

This shows that C increases for *ε*_2_ greater than *ε*_1_. The dielectric *ε*_2_ is now removed and replaced by a metal slab of thickness *d_1_*. It makes no difference where this metal slab is located between the plates. It can be placed to touch either plate *A* or *B*, which can be expressed as Equation (63):

(63) $$C=\varepsilon_{1}S/4\pi \left(d-d_{1}\right).$$

Equation (63) can be derived from Equation (62) if *ε*_2_ is given the value of infinity. In this case, we have the theorem sought:

- A conductor acts like an insulator whose dielectric constant is infinitely large.

### 4.2 Polarization of a Dielectric

**FIGURE 8:**
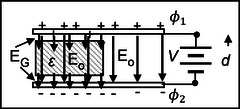
Insertion of a dielectric into an air‐filled condenser creates added charges to its plates, shown here maintained at a fixed potential difference *V* ($\phi_{1}-\phi_{2}$). The relationship between the field in the air gap and the dielectric is given by $E_{G}=\varepsilon E_{o}$.

Consider Figure 8 which depicts a slab (e.g., glass) of dielectric constant *ε* partly inserted between the plates of an air capacitor. The plates are fixed at a distance *d* apart and a constant potential difference $(V=\phi_{1}-\phi_{2}$) is applied to them. Narrow gaps *G* (with *G* << *d*) exist between the dielectric and the surfaces of the two opposing plates– a situation that will negligibly influence this analysis. It is an example of capacitive coupling, which can be exploited to coat electrode surfaces with passive or active materials, for example.

With air (or vacuum) between the plates a uniform electric field E_o_ is produced (Equation 64):

(64) $$E_{0}=\left(\phi_{1}-\phi_{2}\right)/d,$$

corresponding to a surface charge density ±*ω*_o_ on each plate given by Equation (65):

(65) $$4\pi \omega_{o}=E_{o}=\left(\phi_{1}-\phi_{2}\right)/d.$$

The applied potential difference *V* remains constant as the dielectric is inserted, and so the field E_o_ between the plates remains unchanged. However, a new charge density *ω* is created, given by Equation (66):

(66) $$4\pi \omega =\varepsilon E_{o}=\varepsilon \left[\left(\phi_{1}-\phi_{2}\right)/d\right].$$

The action of replacing air with a dielectric slab has thus required the voltage source to supply the following quantity of charge (Equation 67):

(67) $$\omega -\omega_{o}=\left(\varepsilon -1\right)/4\pi \;E_{o}.$$

Because the air gap does not alter the charge capacity of the condenser, the field intensity across it has the value expressed by Equation (68):

(68) $$E_{G}=4\pi \omega =\varepsilon E_{o}.$$

Inside the dielectric slab, however, the field intensity remains at E_o_ because the line integral $\oint E_{s}ds$ of a return path from one plate to the opposite one is zero. At the surface of the dielectric, the field falls in magnitude from *ω*E_o_ to E_o_. From Gauss's law (Equation 25) this is equivalent to the surface of the dielectric carrying a surface charge given by Equation (69):

(69) $$4\pi \omega_{G}=\left(\varepsilon -1\right)E_{o}\cdot n,$$

with **n** the normal vector pointing into the dielectric.

The surface charge density *ω_G_* given by Equation (69) is a manifestation of the induced polarization **P** of the dielectric, expressed as Equation (70):

(70) $$P=\omega_{G}=\left(\left(\varepsilon -1\right)/4\pi \right)E.$$

**FIGURE 9:**
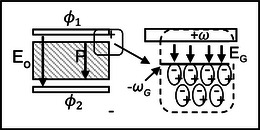
Polarization P of a dielectric induces a surface charge density (+ω) on the positive plate, due to the negative surface charge density at the dielectric's surface. The opposite effect occurs at the negative plate.

As shown in Figure 9, **P** is a vector directed into the dielectric from the electrode surface. The proportionality factor between **P** and **E** is called the dielectric susceptibility χ (Equation 71):

(71) $$\chi =\left(\varepsilon -1\right)/4\pi .$$

The microscopic mechanism involved in the bulk polarization of the dielectric is depicted in Figure 9.

Metals are good conductors because, in their atomic lattices, they have delocalized electrons able to move long distances through this lattice and sustain a constant flow of electrons. A dielectric is an insulator because it lacks delocalized electrons—instead, they are localized and bound quasielastically to positively charged nuclei in their crystal structure. As first proposed by Mossotti [26], in response to an applied electric field, positive and negative charges within individual “corpuscles” of a dielectric are slightly displaced but cannot escape from a corpuscle to form a current flow of electrons. A dipole moment is induced in each corpuscle. In the bulk of the dielectric, the charges of neighboring dipoles cancel, and so no excess charge exists in the dielectric's interior. However, for those corpuscles at the interface of the dielectric and electrode, this charge neutralization can only occur through the induction of counter charges at the electrode's surface, as depicted in Figure 9.

The polarized dielectric slab carries on its opposing faces, distance *d* apart, surface charge densities of equal and opposite magnitude *ω_G_*. This represents a dipole moment of magnitude **m** given by Equation (72):

(72) $$m=\omega_{G}d\;\int \int \;dA=P_{n}d\;\int \int \;dA=P_{n}\;\int \int \int \;dV.$$

The polarization **P** therefore represents the induced dipole moment per unit volume of the dielectric.

### 4.3 Maxwell's Electric Displacement Vector D

Maxwell introduced a new vector **D**, called electric displacement, to replace the vector **E_n_** in Equation (26). It satisfies the stipulation that the integral of the normal component of the vector **D**, extended over a closed surface *S*, or the total electrical displacement through the surface, is equal to the total electric charge enclosed by the surface [8, p. 146]. As the displacement increases from an initial value of zero to its final value P, the amount of electric displacement is defined by Maxwell as the quantity of electric current that crosses a unit of surface area of the dielectric. This, therefore, is the measure of electric polarization [17, p. 65]. Maxwell's conception of electric displacement was influenced by Mossotti's hypothesis [26] of the displacement of charges in corpuscles as depicted in Figure 9. From Equations (27) and (66):

$\mathrm{div}\;E=4\pi \rho_{G}$, so that (Equation 73):

(73) $$D_{n}=\varepsilon E_{n},$$

to give $D_{n}=E_{n}+4\pi P_{n}$, and (Equation 74):

(74) $$P_{n}=\frac{1}{4\pi }\;\left(\varepsilon -1\right)E_{n}\;\left(\mathrm{CGS}\;\mathrm{units}\right);P_{n}=\varepsilon_{o}\;\left(\varepsilon -1\right)E_{n}\;\left(\mathrm{SI}\;\mathrm{units}\right)$$

In Figures 7 and 8, the gap between the electrode plate is assumed to be air or vacuum, but it could be a solid or liquid. The displacement vector **D** can thus be attributed to the following properties:

1. In the bulk interior of an uncharged dielectric div D = 0 everywhere.
2. At the boundary between two insulators the normal component of D is everywhere continuous—there are no sources or sinks of flux (charge).

### 4.4 Boundary Conditions at Dielectric Surfaces

**FIGURE 10:**
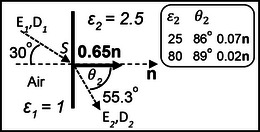
E‐ and D‐lines of force at the boundary surface *S* of air and a dielectric *ε*_2_ according to the law of refraction given by Equation (75).

Properties 1 and 2 listed above for **D**, together with **D** and **E** being continuous everywhere, provide the *law of refraction* for the **D**‐ and **E**‐lines of force at the boundary surface between two ideal dielectrics. 1 and 2 can be written as:

$$\left|D_{1}cos\theta_{1}\right|=\left|D_{2}cos\theta_{2}\right|$$

$$\left|E_{1}cos\theta_{1}\right|=\left|E_{2}cos\theta_{2}\right|$$

where *θ*1 and *θ*1 are the angles, as shown in Figure 10, of the D‐field lines and E‐field lines of force with the normal vector n to the boundary surface S. The tangential components of **E** and **D** at a boundary surface are also continuous, so that with D=εE we have for the law of refraction (Equation 75):

(75) $$\tan\;\theta_{1}/\tan\;\theta_{2}=\varepsilon_{1}/\varepsilon_{2}.$$

An example of the refraction at the interface between air and a polymeric insulator such as polystyrene (dielectric constant value ∼ 2.5) is shown in Figure 10. The E‐ and D‐lines make contact with this interface at 30° to the normal vector at the surface and emerge into the dielectric at an angle of 55.3° to the normal. The normal component of the incident lines of force is 0.87**n** and drops to 0.65**n** inside the dielectric. Tabulated values of the normal components emerging from the dielectric surface are shown in Figure 10 for ethanol (*ε*_2_ = 25) and water (*ε*_2_ = 80). With an increasing dielectric constant value for *ε*_2_ the normal component of **E** and **D** rapidly tends to zero. We know that a conductor (metal) cannot sustain an internal field, so this result provides another way to validate the rule:

- A conductor acts like an insulator whose dielectric constant is infinitely large.

A description is given in Appendix 5 of how these boundary conditions are used to derive the induced dipole moment.

#### 4.4.1 A Point Charge in Front of a Dielectric Surface

As suggested by its name, the law of refraction can be incorporated into the method of images to analyze the effect of a dielectric surface from the perspective of the field of an external point charge. This problem, equivalent to that already solved in section 3.7.1 for the case of a point charge Q in front of a conducting surface, is left as an exercise for interested readers. As a hint for this, place an imaginary charge −$Q^{\prime }$ inside the dielectric *ε*_2_ as a mirror image of the real charge Q, and find the potential at a point on the boundary in terms of the distances from Q and −$Q^{\prime }$. From the perspective of the field inside the dielectric *ε*_2_, what is the effect of charge Q? Extend the situation to include that, apart from air, Q could also reside within a dielectric *ε*_1_. The following relations that satisfy the boundary conditions are expressed as Equations (76) and (77):

(76) $$\left(Q-Q^{\prime }\right)/\left(Q+Q^{\prime }\right)=\varepsilon_{1}/\varepsilon_{2},$$

(77) $$Q^{\prime }=Q\frac{\left(\varepsilon_{2}-\varepsilon_{1}\right)}{\left(\varepsilon_{2}+\varepsilon_{1}\right)}.$$

The situation should also be satisfied that no sources or sinks of electrical flux can exist within the volume occupied by the dielectric. To do this, assume that the dielectric occupies all space and places an imaginary charge $Q^{\prime \prime }$ at the location of Q. The following value for $Q^{\prime \prime }$ satisfies Equations (78):

(78) $$Q^{\prime \prime }=Q+Q^{\prime }=Q\left[2\varepsilon_{2}/\left(\varepsilon_{2}+\varepsilon_{1}\right)\right].$$

In Equation (77), consider the case where the dielectric constant *ε*_2_ increases to a very large value compared with *ε*_1_‐taken to be air (*ε*_1_ = 1). In this case Q and $Q^{\prime }$ become equal in magnitude—exactly the situation shown in Figure 5B. Thus:

- Metals influence the field in air in the same way as a dielectric of infinite dielectric constant.

#### 4.4.2 Induced Polarization of a Dielectric Sphere

Abraham & Föppl [8, p. 163] do not provide scientific references in their textbooks, and so do not mention that the method they teach [8, p. 163] to derive the polarization of a dielectric sphere is essentially the same as that described by Green [27] for the polarization in a magnetic field of an iron sphere in air. As shown in Appendix 5, a spherical particle of dielectric constant *ε_p_* is assumed to be immersed in a medium of dielectric constant ε_m_ that occupies the whole of the rest of space. A uniform field **E**_o_ is assumed to have already been established inside this dielectric medium before inserting the spherical particle, and its induced polarization is evaluated in terms of an assumed induced dipole moment **m**. The result given in Appendix 5 for the induced dipole moment is expressed as Equation (79):

(79) $$m=\varepsilon_{m}R^{3}\left(\frac{\varepsilon_{p}-\varepsilon_{m}}{\varepsilon_{p}+2\varepsilon_{m}}\right)E_{o},$$

The dielectric sphere's polarization **P** is the induced moment per unit volume (Equation 80):

(80) $$P=3/4\pi \;\left[\varepsilon_{m}\left(\frac{\varepsilon_{p}-\varepsilon_{m}}{\varepsilon_{p}+2\varepsilon_{m}}\right)\right]E_{o},$$

The induced dipole moment **m** of a metal sphere in the air is obtained with *ε*_m_ = 1 and *ε_p_* assigned an infinitely high value, and so from Equation (79) we obtain the same result as Equation (58) by using the method of images:

$$m=R^{3}E_{o}$$

- The disturbing effect on field **E** of a conductive sphere may be represented by a double source of moment R^3^ **E** in the center of the sphere. [5, p. 166]

Furthermore, the term in brackets in Equation (80) has a value of around 1 if *ε_m_* = 1 (for air) and if by analogy *ε_p_* is taken to be the relative magnetic permeability *μ_p_* of a magnetic material. Iron has a value of *μ_p_* ∼100 if not pure (5000 if 99.8% pure). This corresponds to Green obtaining for an iron particle a value close to 1 for his magnetic polarizability factor *g* [29, p.61].

#### 4.4.3 Dielectric Sphere in a Field Gradient

Boltzmann derived an expression for the ponderomotive force by first considering *N* point charges clustered tightly around the origin of coordinates *x*, *y*, and *z*. The external field due to these charges is then expressed as the Taylor expansion (Equation 81):

(81) $$E\left(r\right)=E_{o}+{\left(\partial E_{o}/\partial r\right)}_{o}\frac{r}{1!}+{\left(\partial^{2}E_{o}/\partial r^{2}\right)}_{o}\frac{r^{2}}{2!}+\cdots ,$$

where the suffix “0” indicates that the values are taken at the origin. Ignoring the differentials higher than the second order, then for a small enough cluster of charges the field is given to a good approximation by Equation (82):

(82) $$E\left(r\right)=E_{o}+\left(r\cdot \nabla_{o}\right)E_{o}.$$

The ponderomotive (DEP) force acting on this cluster is expressed as Equation (83):

(83) $$F=\sum_{i}^{N}q_{i}E\left(r_{i}\right)=\sum_{i=1}^{i=N}q_{i}+\left(\sum q_{i}r_{i}\cdot \nabla \right)E$$

The electric moment M of the cluster of charges is given by Equation (84):

(84) $$M=\sum_{i=1}^{N}q_{i}r_{i}.$$

Let the total charge $Q=\sum q_{i}$, then (Equation 85):

(85) $$F=QE_{o}+\left(M\cdot \nabla \right)E_{o}.$$

Applying this result to describe the force acting on a small dielectric sphere whose induced moment is given by Equation (79), and, in addition, carries a charge Q, then (Equation 86):

(86) $$F=QE_{o}+\left(m\cdot \nabla \right)E_{o}=QE_{o}+\varepsilon_{m}R^{3}\left(\frac{\varepsilon_{p}-\varepsilon_{m}}{\varepsilon_{p}+2\varepsilon_{m}}\right)\left(E_{o}\cdot \nabla \right)E_{o}$$

This shows that an uncharged dielectric sphere in air ($Q=0;\;\varepsilon_{m}=1)$ is directed by the ponderomotive force toward a location of greater field strength.

### 4.5 Field Energy and the DEP Force

Maxwell shows that the electrostatic energy of the whole field of a system resides in every part of the field where electrical force and electrical displacement occur [17, vol 2, p. 270]. This potential energy, per unit volume, is evaluated over all space using Equation (87):

(87) $$U=\frac{1}{2}\int_{v}E_{m}\cdot D_{m}dv.$$

**E**_m_ is assumed here to have been established in the medium before the insertion of a particle into it. If the electrostatic field is in free space, then **D**_m_ = **E**_m_. Polarization of a particle introduced into the medium produces a (dipole) field that modifies **E**_m_. Designating this modified field at any point as **E**, then the field resulting from the particle polarization is given by $E_{p}=\left(E-E_{m}\right)$. The energy *U*_2_ of the field in this new state is given by Equation (88):

(88) $$U_{m}=\frac{1}{2}\int_{v_{p}+v_{m}}E\cdot Ddv,$$

where *v*_p_ and *v*_m_ are the volumes occupied by the particle and the medium outside the particle, respectively. The change in energy *U* = (*U*_2_ ‐ *U*_m_) is equal to the energy of the particle in the external field E_m_, and is given by Equation (89):

(89) $$U=\frac{1}{2}\int_{v_{p}+v_{m}}\left(E\cdot D-E_{m}\cdot D_{m}\right)dv,$$

This also represents the work performed for the action of inserting the particle into the field **E**_m_. Equation (89) can be written as Equation (90):

(90) $$U=\frac{1}{2}\int_{v_{p}+v_{m}}E\cdot \left(D-D_{m}\right)dv+\frac{1}{2}\int_{v_{p}+v_{m}}\left(E-E_{m}\right)\cdot D_{m}dv.$$

We now assume that the particle carries no net charge and that an external agent (e.g., signal generator) maintains the original charge distributions producing **E**_m_. In this case, the first integral in Equation (90) is zero, which gives Equation (91):

(91) $$\begin{array}{ccc} U & = & \frac{1}{2}\int_{v_{p}+v_{m}}\left(E-E_{m}\right)\cdot D_{m}dv=\frac{1}{2}\int_{v_{p}}\left(E-E_{m}\right)\cdot D_{m}dv\\ & & +\frac{1}{2}\int_{v_{m}}\left(E-E_{m}\right)\cdot D_{m}dv. \end{array}$$

A theorem of vector fields states that the integral over all space of the scalar product of an irrotational vector and a continuous vector is zero. This theorem can be applied here because both $curl\;E_{m}$ and $div\;(D-D_{m})$ are zero. Equation (91) can then be reduced to the single integral, not over all space, but solely over the particle's volume [5, p. 68; 24, p. 113; 28, p. 167] (Equation 92):

(92) $$U=\frac{1}{2}\int_{v_{p}}\left(E\cdot D_{m}-E_{m}\cdot D\right)dv=\frac{1}{2}\int_{v_{p}}\left(\varepsilon_{m}-\varepsilon_{p}\right)E\cdot E_{m}dv.$$

Equation (92) is important for the practice of DEP. It indicates that for *ε_p_* > *ε_m_* we can expect the introduction of the particle into the field to result in a negative value of its potential energy. Work will be required on the particle to withdraw it from the system. Furthermore, the work required will increase more if **E**_m_ is increased, because the particle will attempt to minimize its potential energy further by moving up a field gradient to a greater field value. This describes the action of positive DEP, where a particle is directed toward an electrode edge. On the other hand, if the particle's dielectric constant is less than that of the medium, it will move down a field gradient to search for a field minimum, well away from an electrode edge. Work has been done by the field to achieve this situation, and work will be required by an external agent to push the particle back into a stronger field region of the system composed of medium and electrodes. This describes negative DEP. Furthermore, if a small change δε occurs in the dielectric constant of the system, then $\delta \varepsilon \;E\cdot E_{m}$ will differ very little from $\delta \varepsilon \;E_{m}^{2}$, so that Equation (92) becomes Equation (93):

(93) $$\delta U=\frac{1}{2}\int \delta \varepsilon E_{m}^{2}\;dv.$$

This result shows that a small change in the dielectric constant of the particle relative to that of the suspending medium can be monitored as a change in the DEP force. From Equation (51) that relates the dielectric constant to the fixed dimensions of the system, the change in potential energy is directly related to its capacitance expressed by Equation (94):

(94) $$\delta U=\frac{4\pi d}{2S}\int \delta CE_{m}^{2}\;dv.$$

An increase in capacitance results in a decrease in the potential energy of the system, in accordance with Hatfield's rule that

- The particles move to increase the dielectric capacity of the system.

#### 4.5.1 DEP Response of Latex Beads in an Aqueous Medium

The DEP response of latex beads shown in Figure 1 can now be considered from the perspective of not just the beads but also from the potential energy of the whole system. The planar interdigitated electrode design used in this experiment has adjacent fingers of one electrode interlocking with the fingers of the other electrode. This is depicted in Figure 10A,B, together with the final location of the chains of the beads after their initial levitation from the plane of the electrodes.

**FIGURE 11:**
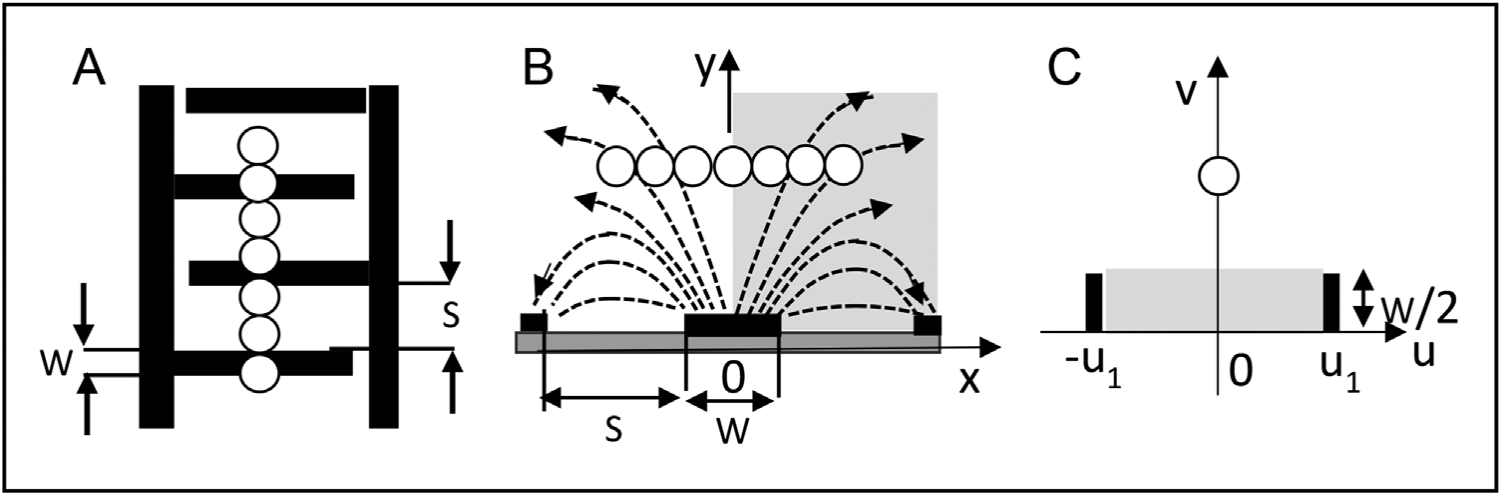
A, End point of the DEP experiment shown in Figure 1, with levitation and chaining of latex beads above interdigitated electrodes. B, Electrodes, lines of electric force and a chain of beads. C, Conformal mapping of the electrodes to a configuration where its capacitance can be determined. The beads have moved away from the strong field between the electrodes.

The width w and pitch s of the electrode fingers affect the capacitance of the electrode system, and calculation of this can be achieved using the method of conformal mapping [29, 30] to convert the planar geometry to the form shown in Figure 11C, and where the capacitance of a pair of electrode fingers can more readily be calculated based on the geometry of a plate capacitor. The latex beads, with a dielectric constant of around 2.5, have clearly moved from the region of high field between the plate electrodes to a location in the weaker fringe field. The volume originally occupied between the electrode plates has been replaced by an equal volume of the aqueous medium of dielectric constant around 80. From Equations (93) and (94) the capacitance of the system has increased, in compliance with Hatfield's rule.

#### 4.5.2 DEP Theory for AC Fields

As well as DC experiments, Boltzmann [2] and Hatfield [6] performed measurements of the ponderomotive (DEP) force at 100 Hz. The DEP behavior shown in Figure 1 was the response to a 1 MHz voltage signal. Electrostatics theory assumes, as the name implies, the static case where charges on an electrode and the field do not depend on time. A dielectric placed between plate electrodes is polarized by the field, and Mossotti's [26] mechanism for this involves the polarization of quasielastically bound charges. Their displacement will exhibit some inertia. If a constant field is suddenly applied the polarization *P* of Equation (74) will not reach its steady state (static) value immediately but will approach this gradually, with a relaxation time that depends on physical quantities associated with molecular structures and their interactions [31, 32]. Displacement *D* will be proportional to *E*, as given by Equation (73) but, because of the phase shift between them, their ratio will depend on frequency. For a periodic field $E=E_{o}\cos\omega t$ the displacement *D* will also be periodic in time but given by Equation (95):

(95) $$D=D_{o}\cos\left(\omega t-\varphi \right)=D_{1}\cos\;\omega t+D_{2}\sin\omega t,$$

where *φ* is the phase shift. This introduces two different dielectric constants, *ε’* and *ε’’*, given by $D_{1}=\varepsilon^{\prime }E_{0}$ and $D_{2}=\varepsilon^{\prime \prime }E_{0}$. The second dielectric constant, *ε’’*, is proportional to the energy loss in dielectrics [33, p.14]. As such, a quantity called the apparent AC conductivity can be defined as $\sigma_{AC}=\varepsilon_{o}\varepsilon^{\prime \prime }\omega$. These relationships can be condensed into the following forms, by introducing a complex dielectric constant and replacing $E=E_{o}\cos\omega t$ (Equation 96):

(96) $$\varepsilon^{*}=\varepsilon^{\prime }+i\varepsilon^{\prime \prime };\;E=E_{o}\;e^{-i\omega t},$$

where $i=\sqrt{-1}$, and considering only the real part of $e^{-i\omega t}$.

For the case of DEP experiments performed using AC voltages, and based on Equations (2), (59), and (79), the familiar expression for the DEP force exerted on a spherical particle is obtained (Equation 97):

(97) $$F_{DEP}=2\pi \varepsilon_{o}\varepsilon_{m}R^{3}\;Re\left[\frac{\varepsilon_{p}^{*}-\varepsilon_{m}^{*}}{\varepsilon_{p}^{*}+2\varepsilon_{m}^{*}}\right]\nabla E_{\mathrm{rms}}^{2}$$

where Re signifies that the real component of the term in brackets (called the Clausius‐Mossotti factor) should be taken.

## 5 Hatfield's Patent and Ph.D. Thesis

### 5.1 The Patent (US 1 498 911)

**FIGURE 12:**
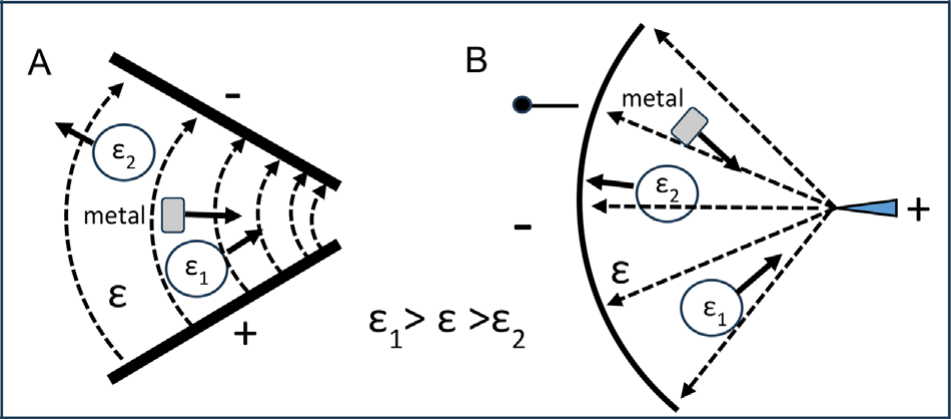
A, Polarized particles move perpendicular to the lines of force generated by nonparallel metal electrodes. B, Polarized particles move parallel to the radial lines of force generated by a point electrode. A particle moves along the direction of the steepest field gradient to a region of stronger or weaker field according to whether its permittivity is greater or less than that (*ε*) of the suspending medium.

This patent can be downloaded from either the US Patent Office or from the European Patent Office (Espacenet) and so there is no need to describe it in detail here. However, the following summary of the rules, shown schematically in Figure 12, and the methods he describes are remarkable. In considering these rules, it should be noted that most particles carry a net surface charge and with a DC potential difference applied to the electrodes they will experience an additional electrophoretic force, additional to the DEP force, that is parallel (not perpendicular) to the field lines of force. With a low frequency AC signal and a field having a steep gradient of its intensity, a charged particle will slowly drift toward a region of higher field intensity. It should also be emphasized that the following rules of DEP do not apply to insulator‐based DEP experiments. One significant difference here being that the field lines of force do not meet an insulating structure at right angles to its surface and that the angle of incidence could alter with time during an experiment.

#### 5.1.1 Schematic Summary of Observations of DEP

To explain how exploitation of his invention may be understood, Hatfield in Figure 1 of his patent draws a series of dotted lines to represent lines of electric force. The lines are shown parallel to one another with the distance between them progressively decreasing towards the right hand. He explains that the strength of an electric field is indicated by the spacing of the lines of force, and so this field becomes progressively stronger from left to right. A practical manifestation of this and schematics of how different small bodies are urged in a direction at right angles to the lines of force are shown in Figure 12A. In Figure 2 the lines of force are represented as radiating out from a point, which may in practice be the section of a knife edge. As shown in Figure 12B, the small bodies are now urged in a direction along the lines of force.

#### 5.1.2 The Methods and Rules of DEP

- Electrodes in the liquid are connected to an electric supply of considerable potential, preferably but not necessarily alternating. An electrostatic field between the electrodes is thereby produced.
- As a rule, direct current is not desirable because electro‐osmose may then interfere with the phenomena described below.
- Suspended particles of greater electrical polarizability than the suspension medium move to the strongest part of the electrostatic field.
- Particles of smaller polarizability than the suspension medium move out of the electrostatic field between the electrodes.
- The rule is that the particles move to increase the dielectric capacity of the system.
- Particles move in the direction of steepest field gradient, as indicated in Figure 12.
- If the insulating particles are replaced by conducting ones, they behave as if their permittivity were very large and always move to the strongest part of the field.
- The electrodes may be plates set parallel to one another and covered with fine metallic points upon the surfaces adjacent to one another. The plates may be 0.25 to 1 mm apart, charged with alternating current of 200 volts, and suitably insulated by making the rest of the chamber of glass or some other insulating material.
- If bare metal electrodes are used, the presence of conducting particles in the suspension gives rise to short circuits if they are permitted to accumulate on the electrodes, since they tend to form chains which finally reach from electrode to electrode and short circuit them. This effect can be avoided by interposing porous separators between the electrodes.
- If one of the conducting boundaries of the electric field is formed by a conducting liquid, which is immiscible with the liquid forming the medium of suspension, then particles of less dielectric polarizability than the medium will tend to be driven into the liquid electrode, because their places in the field are filled by the suspension medium. In doing so, the total electrical capacity of the field has increased.

The caution Hatfield expresses in (b) regarding electroosmotic effects will be appreciated by modern practitioners of DEP. Worthy of note is his observation (i) of the formation of chains of particles (now referred to as pearl chaining) and the use (j) of liquid electrodes which predates by more than 80 years their application by Demiere et al. [34]. His rule (e) that particles move to increase the dielectric capacity of the system is a good indication that he had assimilated the first five chapters of Abraham & Föppl to where it is demonstrated [14, p. 184] that:

The force acting in the nonuniform field seeks to drive the dielectric sphere toward places of greater field strength because now the field energy is reduced by the presence of the sphere. This force is independent of the direction of the field strength.

### 5.2 Hatfield's Ph.D. Thesis [6]

As stated in the introduction, Hatfield's objective was to develop his concept of dielectric separation into a practical method of separating minerals, first in the laboratory and then, if possible and economical, to apply this to the separation of cassiterite from Cornish ore [6, p.12]. Valuable minerals such as tin are normally found as an intimate mixture with an excess of valueless matter that is technically described as gangue. The methods at that time in Cornwall resulted in a loss of about 35% of the tin content of this ore. He explains that physical processes of separation fall naturally into two classes—positive and negative. For positive separation, an applied force acts on the desired constituent of a mixture in one direction, whilst for the unwanted ones the force acts in the opposite direction. In the case of ‘negative’ separation the force acts more strongly for one constituent than the other and so separation depends upon this difference. Magnetic separation can be described as a positive process since all solid substances fall into one of three classes, namely: ferromagnetic, slightly magnetic, and where magnetic properties are only detectable using sensitive instruments and strong magnetic fields. A weak field separates out the first class from the other two, and a strong field the second from the first. Electrostatic separation is differential in character. A mixture of solid particles is brought into momentary contact with a metal electrode charged to a high potential. Electrically conducting particles acquire charge more rapidly than insulating ones and are repelled from the electrode immediately. However, because the cohesive forces between particles increase as the ratio of their surface area to volume, this method only works well when the material is not too finely ground down into a powder. This limitation works against both magnetic and electrostatic separations, because (as the name implies) the pondermotive force increases as a function of particle size.

Hatfield cites Equation (1) derived by Boltzmann as described by Drude [5]. The dielectric constant of minerals varies within the limit of about 3 to 100, and so the differential effect predicted by Equation (1) is clearly far too small to offer an efficient separation process. However, the differential type of electrostatic action may be changed to a positive type by the simple device of suspending the mixture, the constituents of which are to be separated, in a medium whose dielectric constant lies between that of the constituents [6, p.4]. Equation (1) is also an approximation, in that the sphere is assumed to be small in comparison with the rate of change of the field so that the field inside it may be regarded as uniform. In actual practice, we must deal with particles of irregular shape, and frequently of a size which exceeds that permitted by this approximation. “Nevertheless, the application of the above formula yields some numerical results which are instructive, as giving the order of magnitude of the effects to be expected and the nature of their variation with various constants of the apparatus and substances employed” [6, p.5].

The simplest form of apparatus considered by Hatfield for such numerical assessments consisted of a pair of electrodes, formed of a spiral of fine wire for one pole and a fine wire along its axis connected to an alternating voltage supply. Assuming the spiral to be equivalent to a closed conducting cylinder, the electrostatic capacity per unit length can be taken as $K_{1}/\left(2\ln[R_{2}/R_{1}]\right)$ where *R*_1_ is the radius of the axial wire and *R*_2_ is the inner radius of the spiral. The field gradient factor $dE^{2}/ds$, and hence the ponderomotive force P in Equation (1), could thus be calculated as a function of applied field **E**; a range of values for *K*_1_, *K*_2_; particle volume v; wire radii *R*_1_, *R*_2_; particle location [6, pp. 5‐11]. Also considered were counter forces acting against the ponderomotive force (fluid viscous drag; gravity; fluid turbulence caused by the “electric wind”; Joule heating) and practical limitations such as wire diameter and electrode spacing. The results of these assessments are presented graphically in an appendix to his thesis. Measurements of the dielectric constants of the liquids used were made using “the Nernst telephone bridge with resistance compensation”. Determination of the magnitude of fluid turbulence involved an ingenious method of comparing the motion of ‘motes’ in the fluid with that of black dots on white paper fixed to a rotating disc. “As a very rough approximation, the maximum fluid velocity of turbulence might be taken as 1 cm. per second” [6, p. 8].

Hatfield considered other effects that could be predicted from the theory, stated verbatim from his thesis [6, p.12]:

“Drude points out that in a homogeneous field between smooth plates, there is a force tending to move a body, whose dielectric constant differs in either direction from that of the medium, toward the nearest plate. This is due to the distortion of the electric field by the presence of the body. By its presence, a body of lower dielectric constant than the medium tends to spread out the lines of force, and this divergence is non‐symmetrical if the body is nearer one plate than the other. In an infinite homogeneous field, the homogeneity of the field is equally disturbed on either side of the body in the direction of the line of force, but in the case above, the field between the body and the nearest plate is weakened as compared with the field between the body and the further plate. Hence, the body of a lower dielectric constant tends to move that way, while if the dielectric constant of the body is higher than that of the medium, the field between it and the nearest plate is strengthened, and the body also tends to move into it.

It is also evident from the general properties of lines of force, that two nonconductors in a homogeneous field will tend to attract one another if their dielectric constant is greater than that of the medium and to repel one another if it is less. This is readily illustrated by the dissipation of flocks of matter of dielectric constant lower than the liquid.

A further well‐known effect, which has not received an entirely satisfactory explanation, is the rotation of bodies in an electric field (usually known as Quincke's rotations). This can sometimes be observed with particles, at any rate with low‐frequency current (50∼). As far as I know, it has not been observed before with alternating current.”

Although this last paragraph suggests observation of particle rotations in a 50 Hz field, Hatfield does not provide any descriptions or examples of such electrorotation in his thesis.

**FIGURE 13:**
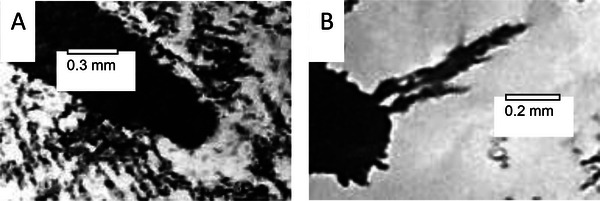
A, Cobalt oxide crystals, suspended in a mixture of nitrobenzene and xylol, repelled by negative DEP from the tip of a needle electrode. B, For a mixture of uranium oxide (UO_2_) and quartz suspended in nitrobenzene, UO_2_ forms chain structures at the electrode tip under the influence of positive DEP. Quartz has been repelled and spatially separated from UO_2_ by negative DEP. Source: Adapted from Hatfield [6, Fig 16].

Two methods of experimentation are described in the thesis. One method used a Zeiss binocular microscope, at 30 times magnification, to find whether certain minerals and other substances are attracted or repelled in an electric field generated between a pair of metal sewing needles mounted in a nonconducting holder. Examples of this are shown in Figure 13 and are discussed in Appendix 5. The point of one needle was broken off and the other one (shown in Figure 13) was bent toward it to give an electrode gap between 1 mm to 2 mm. “With these needles rapid tests could be carried out with a few drops of the suspension in a watch glass under a low power microscope, the holder being grasped in the right hand whilst the watch glass was held in the left” [6, p.13]. Motor‐generators supplied 50 Hz currents at a voltage ranging from 110 to 240 V. For measurements up to ∼1 MHz an oscillating valve generator was used, with the voltage measured using a gold leaf electroscope read with a microscope [6, p.22].

**FIGURE 14:**
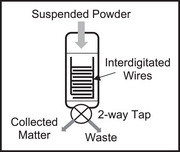
An example of an “electric sieve” tested by Hatfield [6] for small‐scale separation of suspended powdered samples.

The second method performed the actual separation of natural or synthetic materials using wire gratings, described as an “electric sieve”, and validated by chemical analysis. One apparatus tested for small‐scale separations is shown in Figure 14 and consisted of a rectangular glass chamber into which various forms of collecting electrodes could be inserted and connected alternately to opposite poles of an alternating current generator.

The interdigitated electrodes shown in Figure 14 were formed of 42‐gauge (0.08 mm diameter) constantan wire. This cell was filled with the liquid to be used for suspension, the generator was switched on (to first observe the extent of fluid turbulence) and then the powder suspension was added to the reservoir at the top of the chamber. Particles not attracted to the electrodes settled by gravity to the bottom and were drawn off at the tap for collection and examination. The generator was then disconnected, and observation was made of the release and fall of collected material. For some materials, a sharp tapping of the chamber was necessary to cause it to fall from the wires [6, p.14]. This material was also collected and examined.

Various types of electrodes and their configurations were investigated in efforts to scale up the separation process—these are depicted in his patent [1]. In summary, nitrobenzene was found to be the most convenient fluid to use as the suspending medium—also the cheapest. Its dielectric constant is 36, and its boiling point is 209°C. It mixes at room temperature in all proportions with kerosene, though at around 0°C the mutual miscibility is no longer quite complete. It is thus possible to prepare with ease mixtures of any dielectric constant between limits 2 and 36. The confounding influence of the “electric wind” (a term usually used with reference to gases) describes the phenomenon exhibited by an electrified point or edge in an insulating medium. The medium is usually found to be violently repelled where the electric force is strongest, and streams rapidly away from the point. Hatfield investigates this by placing a fine needle with its point downwards as one electrode in a microscope cell filled with nitrobenzene, aniline, or similar fluid. The other electrode was a fine wire gauze placed about a millimeter or less below the needle tip. The convection due to Joule heating then acts upwards, whilst the electric wind from the point is directed downward. The convection of the liquid is easily seen, and at a frequency of 50 Hz and voltage of 200 V is very rapid and directed downward. At 6 kHz it is much weaker, but still downwards, while at around 1 MHz it is gently upward and there appears to be no “electric wind”. Hatfield notes [6, p. 26] that these frequencies correspond to those at which electrolytic polarization is respectively very disturbing, just noticeable, and entirely absent in bridge methods of measuring capacity and resistance by alternating current.

A significant feature observed in the scaling‐up experiments was the formation of chains of crystals along the electric lines of force. “The appearance presented is exactly that of iron filings in the field of a magnet” [6, p.27]. This is now referred to as “pearl chaining” as exemplified in Figure 13A. Chains such as those depicted in Figure 13B persisted after the removal of the voltage. However, the aggregation of quartz, for example, in a liquid of a higher dielectric constant was observed by Hatfield to quickly dissipate by the mutual repulsion of the particles in the field.

Referring to his original objective to improve the separation of cassiterite (stannic chloride) from Cornish ore, he comments that at the start of his research the price of tin, which during the war had reached a very high figure, began to drop and was now below the cost of production. The future of the Cornish mines would probably depend on finding a means to increase extraction efficiency. (Tin mining in Cornwall dates to the Bronze Age, and its last tin mine closed in 1998). He suggests that the best applications of his dielectric separation method would be in the treatment of oxide ores of nickel, cobalt, uranium, vanadium, copper, and so forth, which are not amenable without chemical treatment to the flotation method of separation. As a future experiment, he suggests that the principle of ‘dielectric separation’ can be stated in another form: “If the dielectric in an electric field be made up of parts free to move, and having different dielectric constants, these parts will arrange themselves to make the capacity of the system a maximum” [6, p. 31]. This may have led him to the concept of self‐assembly, or using machines to mass produce other machines, as described in his book “Automaton” [35]. He also hopes to take advantage of the modern development of the thermionic valve to develop new and extremely sensitive means of measuring the dielectric constant.

## 6 Conclusion

Hatfield's patent of 1924 provides a clear and succinct discourse of the electrostatic principles, electrode designs, and modes of operation (e.g., stop‐flow or continuous flow) of relevance to present‐day DEP‐based methods of particle separation. It can readily be reformatted as a DEP tutorial for new contributors to this multidisciplinary subject, especially for those without formal training in electrical technology or physics. The concurrence of the Dublin DEP conference and the patent's 100th centenary offered appropriate timing for this aim. The author first learned of Hatfield's patent in 1993 on receiving the patent examiner's report of an application titled “Apparatus for Separating by Dielectrophoresis”. The questions of how he acquired such deep knowledge and why he was unaware of Willy Wagner's important paper [36] of 1914 arose then and remained until achieving a secondary objective for this article. His internment at the converted racetrack buildings and stables at Ruhleben is the probable reason why his patent [1] does not teach the separation of particles because of their complex conductivity or complex permittivity. Discovery of his Ph.D. thesis resulted in a further objective—only partially achieved. Boltzmann's contributions to DEP were totally unexpected, and further papers he tantalizingly cites have yet to be located.

Lines 24–28 of Hatfield's patent state: “According to this invention the powder the constituents of which are to be separated one from another is suspended in a liquid the dielectric capacity of which lies between that of the constituents of the said powder”. By dielectric capacity, he means dielectric constant. In Appendix 5 this is shown to limit the validity of his claims to high frequencies where $\omega \tau_{MW}>1$, otherwise, the conductivity of the particle and its suspension medium must be considered. In section 5.1.2 describing his rules of DEP, the author has taken the liberty to change dielectric capacity to polarizability. The term polarizability is taken to imply the induced dipole moment per unit volume in the unit field. This then carries Hatfield's rules to our current practice of DEP‐based particle separation—something he would surely have envisaged if he had known of Wagner's paper.

Finally, if I may be allowed to include a personal note: During this exercise, I grew to know and appreciate Dr Henry Stafford Hatfield very well. In his obituary [11], he is described as “an eccentric, ingenious, rebellious and withal buoyant character, he was perforce involved in many adventures and dangers in his long life”. Born on July 12, 1880, in Epsom, England (not far from where this author was born and bred) he would (like me many years later) have been banned from absconding from school lessons to engage in activities at the famous Epsom Downs racecourse. It is surely ironic that Hatfield should formulate DEP‐based particle separation rules at a racecourse near Berlin from which he was banned from absconding!

## Conflicts of Interest

The author declares no conflicts of interest.

## Figures and Tables

**FIGURE A1:**
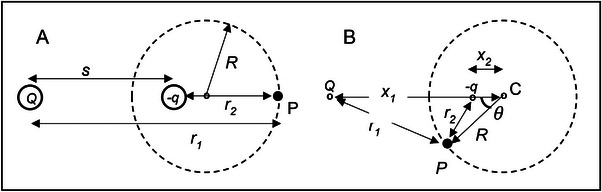
A, Charges Q and ‐*q*, distance *s* apart, with *P* a point of zero potential on an imagined sphere of radius *R*. B, Polar coordinates *R* and θ, and the designated triangles for application of the Cosine Rule.

**FIGURE A2:**
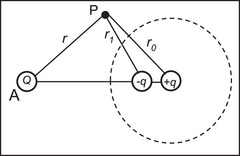
Charge *Q* is located near an insulated metal sphere, of center and radius as shown in Figure A1. Charge +*q* is added to the center of the sphere.

**FIGURE A3:**
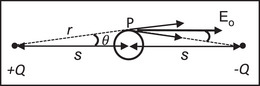
A metal sphere located midway between charges +*Q* and –*Q* spaced 2*s* apart. As *Q* and *s* increase in value, whilst maintaining the local field E_o_, this field becomes more uniform.

## Data Availability

Data sharing is not applicable to this article as no new data were created or analyzed in this study.

## Appendix 1

### 1.1

We wish to generate a spherical surface of constant potential to mimic that of an earthed metal sphere. As shown in Figure A1, consider a positive charge *Q* placed distance *s* from a smaller negative charge −*q*.

The aim is to construct an imaginary sphere that encloses charge ‐*q* such that at any point *P* on its surface, the potential is zero. As *P* is moved around this surface, the distances *r*_1_ and *r*_2_ between *Q* and −*q*, respectively, must adjust to satisfy the condition for zero potential, given by Equation (A1):

(A1)
$$\phi =Q/r_{1}\;-\;q/r_{2}=0.$$

The center of this imaginary sphere is chosen as the origin, *C*, for polar coordinates *R* and *θ*. For the triangles *QPC* and *QPq*, the application of the Cosine Rule of trigonometry gives Equations (A2) and (A3):

(A2)
$$r_{1}^{2}=x_{1}^{2}+R^{2}-2x_{1}Rcos\theta .$$

(A3)
$$r_{2}^{2}=x_{2}^{2}+R^{2}-2x_{2}Rcos\theta .$$

From Equation (A1), the condition to be met for the sphere's surface to be of zero potential is expressed as Equation (A4):

(A4)
$$Q^{2}/q^{2}=r_{1}^{2}/r_{2}^{2}\;,$$

which from Equations (A2) and (A3) is given by Equation (A5):

(A5)
$$\frac{x_{1}^{2}+R^{2}-2x_{1}R\cos\theta }{x_{2}^{2}+R^{2}-2x_{2}R\cos\theta }=\frac{x_{1}}{x_{2}}\;\left[\frac{\left(\frac{R^{2}}{x_{1}}+x_{1}-2R\cos\theta \right)}{\left(\frac{R^{2}}{x_{2}}+x_{2}-2R\cos\theta \right)}\right].$$

This equality is met for all values of the polar angle *θ* by Equations (A6) and (A7):

(A6)
$$x_{2}=R^{2}/x_{1}\;,$$

(A7)
$$x_{2}=Q^{2}/q^{2}\;.$$

Equations (A6) informs us that with a steady increase of the distance *x*_1_ of charge *Q* from the sphere the location (*x*_2_) of the image charge *‐q* moves steadily closer to the center *C* of the circle. Equations (A6) also informs us that if the separation distance *x*_1_ and the magnitude of charge *Q* are both increased at the same time, the image charge *‐q* reduces in magnitude. Also, in the limit as Q and the distance of separation tend to infinity—*q* tends to a point charge located at the center of the sphere.

## Appendix 2

### 2.1

**Method of Images: Point Charge and Grounded Metal Sphere**

The results obtained in Appendix 1 can now be used in the thought experiment where the imaginary sphere is removed, and in its place a charge *‐q* is placed at a distance *x*_2_ from its imagined center *C*, as indicated in Figure A1.B. The value of this charge is given by Equations (A6) and (A7) as Equation (A8):

(A8)
$$-q=-Q\;\sqrt{\frac{x_{2}}{x_{1}}}=-\;QR/x_{1}=-QR/s,$$

where *s* is the distance set between the external charge *Q* and the center of the metal sphere. The location, *x*_2_, of this charge, is given by Equation (A7), and so is set along the axis between *Q* and *C*, at a distance given by Equation (A9):

(A9)
$$x_{2}=R^{2}/s\;.$$

The field of this image charge set at distance *x*_2_, together with the field of the external charge *Q*, produces the required equipotential contour of a metal sphere of radius *R* with center *C*. Outside the surface of the effectively earthed metal sphere only one charge exists, namely *Q*, with the potential at any radial point *r* point from it given by Equation (A10):

(A10)
$$\phi =Q/r\;-\;q/r_{q}=Q/r\;-QR/sr_{q},$$

where *r_q_* is the distance from −*q*_1_ to the point where the potential is to be evaluated.

## Appendix 3

### 3.1

**Method of Images: Point Charge and Insulated Metal Sphere**

A metal sphere is insulated (i.e., protected from being electrified by an external source) and is not connected to the earth's potential. It carries no net charge. Charge *Q* is brought up to it as shown in Figure A2. A charge +*q* must now be placed at the center of the equipotential spherical surface described in Appendix A.

Adding +*q* ensures that the sphere remains without a net charge, whilst placing +*q* at the sphere's center will maintain a constant potential of +*q*/*R* on its surface. The potential at any point *P* outside the sphere is then given by:

$$\phi =Q/r+q/r_{0}-qr_{1}$$

where *r*_o_ is the radial distance to *P* from the sphere's center.

Referring to Equation (A8), which gives the value for *q*, the self‐potential of the imaginary sphere's surface (i.e., in the absence of *Q*) is given by:

$$\;\phi_{\mathrm{surface}}=+q/R=Q/s$$

This is equal to the potential of *Q* when it is located at the center of the sphere.

## Appendix 4

### 4.1

**Method of Images: Metal Sphere in a Uniform Field**

In Figure A3 consider the situation where a charge *Q* is placed distance *s* from the center of a metal sphere. It creates an image charge −*q* inside the sphere. Another charge −*Q* is placed distance *s* on the other side of the sphere and creates an image charge +*q*. The magnitudes of the two charges *Q* are increased in magnitude and moved away at the same time keeping the product *Qq* fixed. As *Q* and *d* approach infinite values, the angle *θ* approaches zero and the external field E_o_ becomes uniform in the vicinity of the sphere. As described in Appendix 1, the image charges +*q* and −*q* form a point dipole moment at the center of the sphere.

Based on the derivation of Equation (87), the potential near the surface of the metal sphere is then given by using Equation (A11):

(A11)
$$\phi =-E_{o}\left[r-\frac{R^{3}}{r^{2}}\right]\cos\theta ,$$

to show that it comprises a component due to the potential of the uniform field plus that due to the induced dipole moment **m** = **E**_o_ *R* ^3^.

## Appendix 5

### 5.1

Induced polarization of a dielectric sphere

A uniform field **E**_o_ (directed along the positive *x*‐axis) is assumed to exist in a dielectric medium having a dielectric constant *ε_m_*. A spherical particle of dielectric constant *ε_p_* is now inserted into this medium. The task is to deduce the form of particle polarization whose field, when superposed onto **E**_o_, produces a resultant potential *ϕ* that satisfies the boundary conditions described in section 4.4:

- On either side of the sphere's surface the normal component of the gradient of *ϕ* changes such that ε(∂ϕ/∂n) remains constant,
- *ϕ* is continuous across this boundary defined by the sphere's surface,
- In all the space *ϕ* satisfies Laplace's equation (∇^2^ *ϕ* = 0).

The condition is also made that

(iv) At distances far beyond the sphere *ϕ* = ‐*Ex*.

If the sphere consists of an isotropic and homogeneous dielectric, it will be homogeneously polarized by the external field **E**_o_ to create an internal field **E**_i_ symmetric about the *x*‐axis given as Equation (A12):

(A12)
$$\phi_{i}=-E_{i}x=E_{i}\;r\;cos\theta \;\left(\mathrm{polar}\;\mathrm{coordinates},\;x=r\;\cos\theta \right).$$

This polarization is represented as an induced dipole of moment **m** = *k* **E**_o_ located at the sphere's center. As a result of spherical geometry and assuming an isotropic dielectric constant, this induced dipole moment will be aligned with the external field. Thus, for all space outside the sphere, the field created by this dipole is superposed onto the field **E**_o_, to give Equation (A13):

(A13)
$$\phi_{o}=-E_{o}x+E_{o}\left[k/\varepsilon_{m}r^{2}\right]\cdot \;x/r=-E_{o}\;cos\theta \left(r-\frac{k}{\left(\varepsilon_{m}r^{2}\right)}\right).$$

Equations (A12) and (A13) satisfy condition (iii), whilst boundary conditions (i) and (ii) require:

$\phi_{i}=\phi_{o}\;\mathrm{and}\;\varepsilon_{p}\;\frac{\partial \phi_{i}}{\partial r}=\varepsilon_{m}\;\frac{\partial \phi_{o}}{\partial r},\;\mathrm{when}\;r\;=\;R.$

$\varphi_{i}=\varphi_{o}\;\mathrm{and}\;\varepsilon_{p}\frac{\partial \varphi_{i}}{\partial r}=\varepsilon_{m}\frac{\partial \varphi_{o}}{\partial r},\mathrm{when}\;r=R.$

Thus:

$$E_{i}=E_{o}\;\left(1-\frac{k}{\left(\varepsilon_{m}R^{3}\right)}\right)\;\mathrm{and}\;\varepsilon_{p}E_{i}=\varepsilon_{m}\;E_{o}\left(1+\frac{2k}{\left(\varepsilon_{m}R^{3}\right)}\right)$$

Solving for *k* we obtain the sphere's induced dipole moment (Equation A14):

(A14)
$$m=k\;E_{o}=\varepsilon_{m}\;R^{3}\left(\frac{\varepsilon_{p}-\varepsilon_{m}}{\varepsilon_{p}+2\varepsilon_{m}}\right)E_{o}.$$

Equation (A12) assumes that the sphere comprises an ideal dielectric. Classical electrostatics considers only two types of substance—those that behave as either a perfect dielectric (insulator) or as a perfect conductor [e.g., 7]. When a field is applied to a dielectric there is a sudden displacement of bound charges opposed by a force of electric elasticity [17, p. 69]. The extent of electric displacement is determined by its dielectric constant *ε*. When the field is removed, the elasticity forces the displaced charges back to their original positions. Thus, when an ideal dielectric is subjected to an alternating field, energy is alternately stored and returned by the dielectric—there is no net loss of electric energy. Electric elasticity is continually overcome in a conductor and so a current of true conduction flow is established—determined by its specific conductivity *σ*. When the field is removed, this current simply ceases and energy is irreversibly lost as heat. However, Maxwell considered there to be very few cases of an ideal dielectric, and that electric displacement and conduction “are going on at the same time” [17, p.450]. There is an electrical phase difference between these two processes so it is mathematically convenient to assign to the conductivity of the particle and its surrounding medium a complex value given by Equation (A15):

(A15)
$$\sigma^{*}=\sigma_{c}+i\omega \varepsilon_{o}\varepsilon_{r},$$

where *σ_c_* is the steady state (DC) conductivity, *ε*_r_ is the relative permittivity, *ω* is the radian frequency of the field, and *i* = √‐1 [36]. The apparent AC conductivity term is then given as $\sigma_{AC}=\varepsilon_{o}\;\varepsilon^{\prime \prime }\omega$. The boundary condition remains that on either side of the sphere's surface, the normal component of the gradient of *ϕ* changes such that ε(∂ϕ/∂n) remains constant, but now *ε_p_* and *ε_m_* are complex quantities as described by Equation (97). To account for the conductivity an additional boundary condition is required, namely:

(v) Across the boundary normal to the sphere's surface, the current density (J = *σ*E) is continuous.

At the instant when the field is applied, it has the same magnitude on each side of the boundary interface but at each time interval, because the conductivity of the sphere differs from that of the surrounding medium, different amounts of charge are carried to the outer surface from that which is carried away from the inner surface. An accumulation of surface charge commences and continues until the internal field adjusts to where the same amounts of charge are carried to and away from the interface.

The accumulation of charges responsible for the induced dipole moment **m** is referred to as Maxwell‐Wagner interfacial polarization, characterized by a relaxation time given by Equation (A16) [36, 37]:

(A16)
$$\tau_{MW}=\varepsilon_{o}\;\frac{\varepsilon_{p}+2\varepsilon_{m}}{\sigma_{p}+2\sigma_{m}}.$$

At frequencies where $\omega \tau_{MW}<1$, the DEP response (positive or negative) depends solely on the particle's effective conductivity, *σ_p_*, and that of the surrounding medium, *σ_m_*. It is positive for *σ_p_* > *σ_m_* and negative for *σ_p_* < *σ_m_* [38, 39]. Only at higher frequencies, with $\omega \tau_{MW}>1,$ does the DEP response depend solely on the displacement currents and hence the dielectric constant values. At intermediate frequencies, the DEP response depends on the conductivity as well as the dielectric constant values.

Finally, consider Hatfield's experiment depicted in Figure 13B, which indicated that quartz crystals exhibited negative DEP when suspended as a mixture with uranium oxide in nitrobenzene. Quartz has conductivity and dielectric constant values (∼10^−13^ S/m, ∼20 [40]) both smaller than those for nitrobenzene (∼1.5 × 10^−8^ S/m, 36 [41]). From Equation (A16) this gives, at 100 Hz, $\omega \tau_{MW}\approx 32.$ This suggests that, as supposed by Hatfield, the negative DEP response shown in Figure 13B for quartz particles resulted primarily from their dielectric constant being less than for nitrobenzene. The positive DEP response exhibited by Hatfield's uranium oxide crystals suggests that they had a dielectric constant value greater than 36.
